# Novel *Pfk13* and *Pfubp1* genotypes in African *Plasmodium falciparum* isolates exhibiting reduced susceptibility to the antimalarials artemisinin and lumefantrine

**DOI:** 10.1128/mbio.03676-25

**Published:** 2026-02-25

**Authors:** Sade Pratt, Donelly A. van Schalkwyk, Lindsay Stewart, Emma Filtenborg Hocke, Samirah Mannan, Charlotte Berry, Julian Muwanguzi-Karugaba, Debbie Nolder, Claire Rogers, Peter L. Chiodini, Ryan C. Henrici, Sophie Moss, Nina Billows, Jody Phelan, Taane G. Clark, Susana Campino, Colin J. Sutherland

**Affiliations:** 1 Department of Infection Biology, London School of Hygiene & Tropical Medicine 4906, London, United Kingdom; 2 Department of Immunology and Microbiology, Centre for Translational Medicine and Parasitology, University of Copenhagen 4321, Copenhagen, Denmark; 3 UK Health Security Agency Malaria Reference Laboratory, London School of Hygiene & Tropical Medicine 4906, London, United Kingdom; 4 Perelman School of Medicine Center for Global Health, University of Pennsylvania, Philadelphia, Pennsylvania, USA; The George Washington University Milken Institute of Public Health, Washington, DC, USA

**Keywords:** antimalarial agents, drug resistance mechanisms, drug susceptibility testing, artemisinin combination therapy

## Abstract

Recent evidence indicates that the clinical efficacy of the antimalarial combination artemether-lumefantrine may be compromised by emerging *Plasmodium falciparum* populations with reduced susceptibility to both components in some parts of East Africa. *In vitro s*tudies of parasites of Ugandan origin collected in the field and from imported cases in European travelers suggest that partial resistance to the artemisinin component, mediated chiefly by genetic variants in the kelch propeller domain of the *pfk13* locus, is arising in concert with increased tolerance of lumefantrine. Genetic determinants of this newly recognized phenotype are unknown. Previous phenotypic and genotypic characterization of six 2022 Ugandan isolates from UK travelers identified two lines, HL2208 and HL2210, with significantly reduced *in vitro* susceptibility to both artemisinin and lumefantrine and variant alleles of interest at resistance-associated loci. In this study, phenotype-genotype associations are evaluated among an extended panel of 50 *P. falciparum* isolates collected from UK travelers since 2012. Parasites with reduced ring-stage susceptibility to artemisinin *in vitro* carrying newly identified or previously established genetic variants in propeller and/or non-propeller domains of *pfk13* came from Kenya, Uganda, Zambia, and Namibia. Variant alleles of *pfap2μ*, *pfcoronin,* or *pfubp1* were also identified in some parasite lines. Among 39 evaluable *P. falciparum* isolates from 15 different countries tested since 2012, six of the ten least susceptible to lumefantrine were from individuals who had traveled to Uganda. *Pfk13-*dependent and -independent partial artemisinin resistance has arisen in multiple African settings, providing an opportunity for the development of reduced parasite susceptibility to the partner drug lumefantrine.

**IMPORTANCE:**

In our studies of UK travelers returning from Africa with malaria, we have encountered a small number of cases where standard combination drugs have failed to completely clear the infection. In this study, we present new assessments of the effectiveness of the major drugs in use against such parasites and link these findings to the genetic profiles of the parasites causing each infection. We identify new genetic types in some of these patient samples that may provide new markers for monitoring malaria drug resistance in African communities.

## INTRODUCTION

The widespread use of artemisinin-based combination therapy (ACT) has been central to the successful treatment of clinical malaria in the 21st century, contributing to an estimated 12.7 million malaria deaths averted between 2000 and 2023 (1). The emergence of *Plasmodium falciparum* with delayed parasite clearance *in vivo* following artesunate monotherapy, seen in the Greater Mekong sub-region (2, 3), was the first indication that ACTs were at risk due to the evolution of parasite resistance. This emergence was associated with specific mutations in the propeller domain region of the gene encoding the *P. falciparum* kelch protein on chromosome 13 (PfK13) (4). These variants arose more than once in Southeast Asia under artemisinin selection, rather than spreading from a single focus (5). A similar phenomenon has more recently been recognized in Africa, where K13 propeller domain variants have arisen *de novo* in Rwanda, Uganda, and Ethiopia (6–10). Of particular concern is the recent finding that the presence of the A675V *pfk13* variant was associated with reduced clinical efficacy of parenteral artesunate, followed by oral artemether-lumefantrine (AL) among hospitalized Ugandan children with complicated *P. falciparum* malaria (11). However, treatment failures following ACT treatment of African *P. falciparum* have also been reported in the absence of associated *pfk13* mutations, suggesting involvement of other loci, of non-parasite factors (12–14), or of waning partner drug effectiveness (15). Variants at additional loci, including *pfap2μ*, *pfubp1,* and *pfcoronin,* have also been implicated in reduced artemisinin and/or partner drug susceptibility in human infections with African *P. falciparum* (15, 16), in studies of artemisinin-selected murine malaria (17, 18), and in genetically engineered variants of common laboratory *P. falciparum* cell lines (19–21). Recent functional studies (22) and genetic characterization of *Plasmodium yoelii* parasites selected for survival in mefloquine-treated mice (23) have further implicated modulation of the deubiquitinating activity of *Pf*UBP-1 in reduced parasite susceptibility to several drugs in addition to artemisinin, including mefloquine and lumefantrine. These findings and our growing understanding of the role of the UBP-1 hydrolase in ring-stage endocytosis (19, 24) suggest that monitoring variation in the *pfubp1* locus remains an important aspect of *P. falciparum* genetic surveillance in Africa.

Recent evidence from *in vitro s*tudies of *P. falciparum* of Ugandan origin collected in the field and from imported cases in European travelers suggests that partial ring-stage resistance to the artemisinin component, mediated chiefly by genetic variants in the kelch propeller domain of the *pfk13* locus, may be arising in concert with increasing tolerance of lumefantrine. Tumwebaze et al*.* (8) compared lumefantrine susceptibility of 49 isolates collected in northern Uganda in 2021, a time when this region was considered to be the origin of emerging partial artemisinin resistance, with *in vitro* lumefantrine susceptibility data for 392 isolates collected between 2016 and 2019 in two other Ugandan districts (25). A significantly higher median half-maximal effective concentration (EC_50_) was observed in the 2021 data set compared to the earlier isolates (16.9 vs 5.1 nM). These findings were supported by an investigation of six *in vitro* parasite lines established from UK travelers returning from Uganda with *P. falciparum* malaria in 2022, two of whom had failed treatment with AL (15). The *in vitro* lines derived from these two travelers, HL2208 and HL2210, displayed significantly reduced *in vitro* susceptibility to both artemisinin and lumefantrine, but only HL2210 harbored a validated resistance-associated polymorphism in the *pfk13* propeller domain, the dominant Ugandan variant Ala675Val. However, each of these lines carried previously undescribed variants of *pfk13* encoding a single amino acid change upstream of the propeller domain: Thr349Ile in HL2208 and Leu145Val in HL2210. Also of note were novel non-synonymous substitutions in *pfubp1*; Tyr1530Cys in HL2210 and Arg3113Leu in HL2208.

Studies to date are consistent with the hypothesis that phenotypes of reduced susceptibility to artemisinin and lumefantrine are emerging together in Uganda under drug pressure, due to the widespread use of AL. The genetic determinants of the newly recognized lumefantrine susceptibility phenotype in Uganda are unknown. As well, the geographical boundaries of this phenotype are uncertain given few recent studies of *in vitro* susceptibility for *P. falciparum* isolates from other African countries, particularly those with high *Plasmodium* burden. In this study, *in vitro* susceptibility phenotypes and genotypes at five loci of interest are evaluated in an extended panel of 50 *P. falciparum in vitro* isolates from UK travelers, of which 22 were collected since May 2022. Each patient had entered the UK from the African continent. Variant alleles of *pfap2μ*, *pfcoronin,* and *pfubp1* were identified among the evaluated parasite lines. Newly identified as well as previously established genetic variants are described in both propeller and non-propeller domains of *pfk13* in parasites with reduced *in vitro* artemisinin susceptibility from Kenya, Uganda, Zambia, and Namibia.

## MATERIALS AND METHODS

### Selection of *P. falciparum* isolates for *in vitro* studies

Samples were selected for adaptation based on evolving research priorities at different time points, following non-systematic, study-specific criteria. From 2012 to 2014, parasite lines were established in partnership with the Hospital for Tropical Diseases, London, and the Medicines for Malaria Venture to provide 21st-century *in vitro* cultures for studies of susceptibility to both established and investigational anti-malarial drugs (26). From 2015 to 2016, the Malaria Reference Laboratory (MRL) focused its activity on a small number of AL treatment failures that had been observed among UK travelers (14). From 2019 to 2022, parasites from patients with negative HRP2-based rapid diagnostic tests were prioritized for the study of deletions in the *pfhrp2* and *pfhrp3* genes (27). Also prioritized during this period were parasites of Nigerian origin carrying emerging genotypes at the *pfdhps* locus that may alter the effectiveness of chemoprevention with anti-folate combinations (28). From 2020 to 2024, UKRI funding was provided specifically to investigate the susceptibility of African parasites to artemisinin and lumefantrine. During this period, parasites from documented AL-treatment failures among UK travelers returning from across Africa were culture-adapted, but Uganda was a particular focus, being a popular destination for UK travelers and a site of emerging loss of susceptibility to both artemisinin and lumefantrine (6, 8, 15). Other factors that affected the decision to attempt culture adaptation included sample quality, sample volume, time elapsed since collection, availability of staff, availability of containment laboratory cabinet space, and availability of human serum and other culture medium components.

Collection of samples from patients attending the Hospital for Tropical Diseases, London, from 2012 to 2017 was approved by the Research Ethics Committee of the University College London Hospitals (Application number: 07/Q0505/60). Culture adaptation of isolates from the UK MRL 2018 to 2025 was approved by the LSHTM Research Ethics Committee (reference: 14710) and the National Health Service (NHS), London-Chelsea Research Ethics Committee (reference: 18/LO/0738).

### Parasite isolation and adaptation to culture

Parasite cultures were initiated from blood samples referred by sending laboratories to the UK Health Security Agency MRL, London School of Hygiene and Tropical Medicine, UK, in potassium-EDTA tubes. Long-term adaptation to *in vitro* cultivation in medium supplemented with human serum was performed as previously described (15).

### *In vitro* drug susceptibility

Parasite lines were tested for susceptibility to lumefantrine and chloroquine using the 72-h dose-response assay with SYBR Green I readout, in medium supplemented with 2% (vol/vol) human AB serum and 5 g/L Albumax II (Thermo Fisher Scientific, 11021045). Artemisinin susceptibility was determined by the ring-stage survival assay (RSA) using flow cytometry to estimate the relative survival of synchronized ring-stage cultures of each HL line following a 4-h exposure to 700 nM DHA, normalized to survival of the same parasite population after 4-h exposure to DMSO (15).

### Direct sequencing of PCR amplicons

The four loci of major interest, *pfk13, pfap2μ*, *pfcoronin,* and *pfubp1*, plus *pfmdr1*, were amplified by conventional PCR and sequenced directly as previously described (15). For *pfubp1*, two variable regions (fragment I and fragment IV) previously described by van Schalkwyk et al*.* (15) were again sequenced. Two additional fragments were also amplified and sequenced (fragments II and III; see Results). These two fragments were included to capture additional variant residues described by Casanova et al*.* (29): Lys1914Asn, Glu1915Lys, and Arg2238Lys.

Amplification for fragment II (*pfubp1* codons 1893–2339) used forward primer 5′-GGACAGCAATATGTATGCTG-3′ and reverse primer 5′-CCAGCCAAAGATATATTCGAC-3′, with cycling conditions of 94°C for 3 min, followed by 40 cycles of 94°C for 30 s/56°C for 45 s/65°C for 2 min 30 s and a final polishing step of 65°C for 5 min. This generated a DNA product of expected size 1,419 bp, based on the reference sequence.

Amplification for fragment III (*pfubp1* codons 2519–2847) used forward primer 5’-TAATCAAATGCTTCCGGGTG-3’ and reverse primer 5’-CGGAAGATGATAAGAAAGGATC-3′, with cycling conditions of 94°C for 3 min, followed by 40 cycles of 94°C for 30 s/56°C for 45 s/65°C for 2 min and a final step of 65°C for 5 min. This generated a DNA product of 1,023 bp.

*pfcrt* genotypes at codons 72–76 and *pfmdr1* gene copy number were assessed by established qPCR methods (16, 30).

### Whole-genome sequencing analysis

Whole-genome sequences were determined from DNA extracted from cultured parasite lines using the Illumina NovaSeq 6000 (Eurofins Genomics, Wolverhampton, UK). The raw sequencing FASTQ files were filtered and processed using the Malaria Profiler tool (https://bioinformatics.lshtm.ac.uk/malaria-profiler/) to identify variants (31). Within this pipeline, raw Illumina reads are trimmed (trimmomatic), mapped to the PF3D7v3 reference genome (bwa-mem), and variants in validated and putative drug-resistance genes are called (*GATK*). High-quality variants with adequate coverage (depth > 5-fold) are reported, including validated drug-resistance markers and other mutations within genes implicated in drug-resistance genes.

### Statistical methods

Data tabulations and analyses were carried out in Excel (Microsoft Corp., Redmond, WA, USA), GraphPad Prism 10 (Dotmatics, Boston, MA, USA), and Stata 18 (StataCorp LLC, College Station, TX, USA). The non-parametric Wilcoxon rank sum test was deployed to compare continuous variables between groups. Dichotomous variables were compared by generating an odds ratio (OR) with 95% CI and significance testing using the χ^2^ distribution.

## RESULTS

### Adaptation of clinical isolates to *in vitro* culture

From 2012 to 2024, 50 imported isolates of *P. falciparum* received by the UK HSA MRL were successfully adapted to long-term *in vitro* culture, each receiving an HL identification number beginning with two digits denoting the year of isolation (Table 1).

**TABLE 1:** Origin and treatment outcomes for 48 *P. falciparum* isolates adapted to *in vitro* culture

Serial	Culture line ID	Year isolated	Countries traveled to	Regimen if known^*a*^	Treatment failure	Described
1	HL1204	2012	Kenya	pART/AL	–	(26)
2	HL1205	2012	Nigeria	pART/AL	–	(26)
3	HL1209	2012	South Sudan	pART/AL	–	(26)
4	HL1210	2012	Ghana	AL	–	(26)
5	HL1211	2012	Ghana	AL	Y	(26)
6	HL1212	2012	Nigeria	–^*c*^	–	(26)
7	HL1214	2012	Burkina Faso	AL	–	(26)
8	HL1301	2013	Equatorial Guinea		–	
9	HL1402	2014	Kenya		–	
10	HL1502	2015	Sierra Leone		–	
11	HL1601	2016	Uganda	AL	Y	(12)
12	HL1901	2019	Angola	AL	Y	
13	HL1902	2019	Kenya		–	
14	HL1904	2019	Chad/Sudan		–	
15	HL1905	2019	Kenya		–	
16	HL1906	2019	Guinea		–	
17	HL2000	2020	Nigeria		–	
18	HL2001	2020	Nigeria		–	
19	HL2002	2020	Sierra Leone		Y	
20	HL2004	2020	Sudan		–	(27)
21	HL2102	2021	Sudan	AL	–	
22	HL2103	2021	Zambia	AL	–	
23	HL2104	2021	Sierra Leone	AL	Y	
24	HL2105	2021	Ghana	AL	–	
25	HL2201	2022	Angola	AL	–	
26	HL2202	2022	Angola	AL	–	
27	HL2203	2022	Nigeria	AL	–	
28	HL2204	2022	Ghana	AL	–	
29	HL2205	2022	Kenya	AL	–	
30	HL2206	2022	Uganda	AL	–	(15)
31	HL2207	2022	Nigeria	AL	–	
32	HL2208	2022	Uganda	AL	Y	(15)
33	HL2209	2022	Nigeria	AL	–	
34	HL2210	2022	Uganda	AL	Y	(15)
35	HL2211	2022	Nigeria	AL	–	
36	HL2212	2022	Uganda	AL	–	(15)
37	HL2213	2022	Uganda	AL	–	(15)
38	HL2214	2022	Uganda	AL	–	(15)
39	HL2301	2023	Côtes d'Ivoire	AL	–	
40	HL2302	2023	Uganda	AL	–	
41	HL2303	2023	Uganda	AL	–	
42	HL2304	2023	Zambia	AL	Y	
43	HL2305	2023	Namibia	AL	Y	
44	HL2306	2023	South Africa	AL	–	
45	HL2307	2023	Sierra Leone	AL	Y^*b*^	
46	HL2308	2023	Sierra Leone	AL	Y	
47	HL2309	2023	Uganda	AL	–	
48	HL2310	2023	Uganda	AL	–	
49	HL2401	2024	Uganda	AL	Y	
50	HL2402	2024	Malawi	AL	Y	

^*a*^ In UK hospitals, some patients commence treatment for falciparum malaria with i.v. artesunate before changing to oral AL. We cannot precisely indicate in which cases this strategy was used.

^*b*^ HL2307 is a pre-failure isolate from the same individual as HL2308, which came from recrudescence 3 weeks later.

^*c*^ “–” indicates missing data; treatment given and therapeutic outcomes are not routinely reported.

### Determination of artemisinin susceptibility in HL-series parasite lines

**Fig 1:**
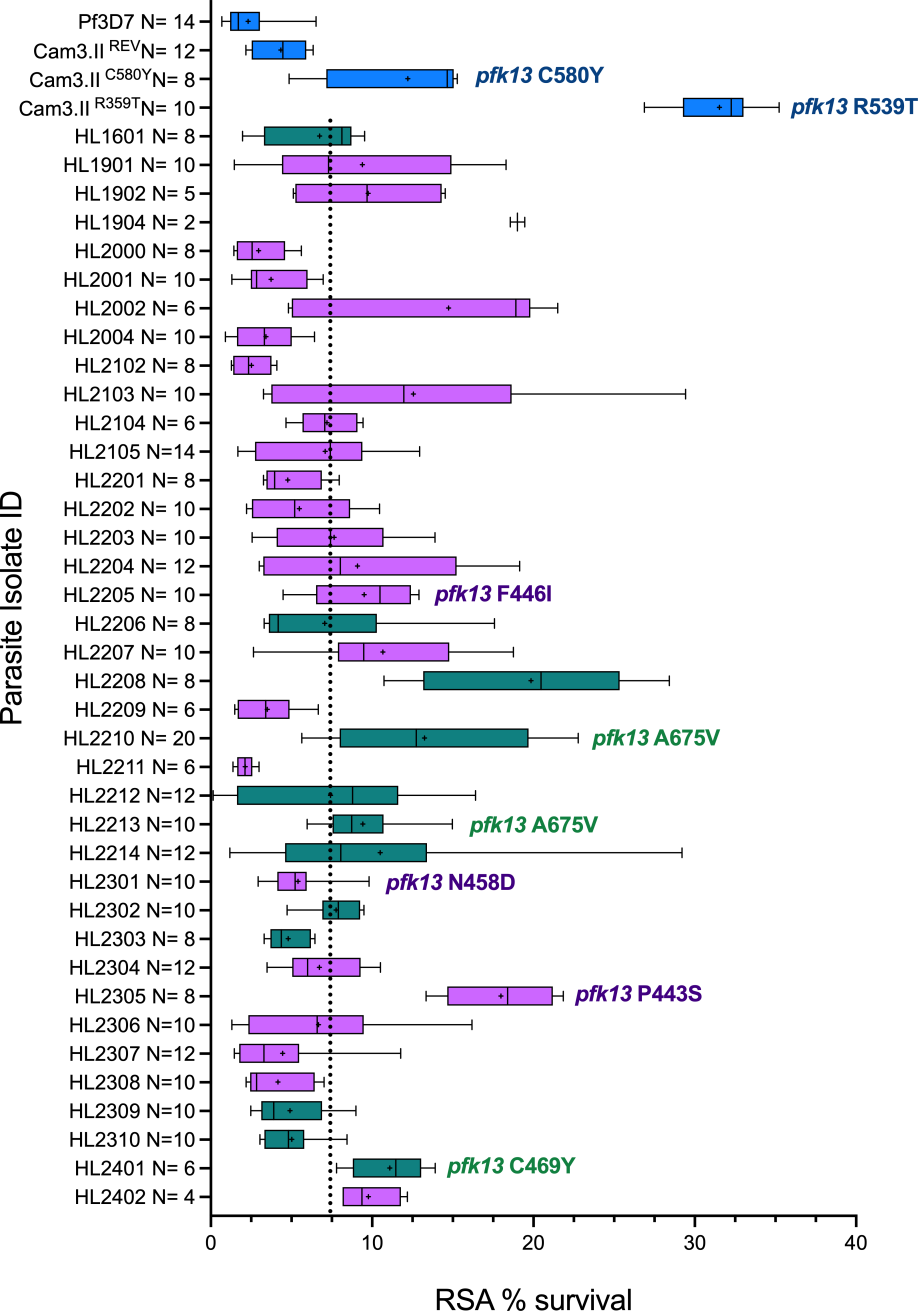
Artemisinin susceptibility of 38 clinical isolates and four comparator lines. Relative survival in the RSA (700 nM DHA, 4 h pulse). Ugandan lines are shown in teal, remaining African lines are lavender, comparator lines are in blue at the top of the figure. Boxes represent interquartile range around the median of all biological and technical replicates. Whiskers represent 95% confidence interval around the mean of replicates, indicated by **+**. Denominators for each isolate are shown onthe *y*-axis. Vertical dotted line indicates unweighted median relative survival among all 38 HL lines of 7.33%. K13 propeller domain genotypes of interest are shown. Five single-point estimates from HL1902, HL2002, and HL2105 were above 40% survival and were discarded.

Susceptibility data for at least one of the antimalarials dihydroartemisinin and lumefantrine were obtained for 40 of the 50 HL lines as shown (Table 2). Those not tested included isolates prioritized for studies unrelated to ACT efficacy. Thirty-eight of 40 clinical isolates were tested in the *ex vivo* RSA, examining ring-stage parasite survival after a physiologic brief exposure to dihydroartemisinin (15, 32) (Fig. 1). Survival was benchmarked against the laboratory clones 3D7 (*pfkelch13* WT) and isogenic Cambodian isolates with the R539T, C580Y, or wild-type *pfkelch13* alleles; RSA survival for all four benchmark parasite lines aligned with published values (Table 2). EC_50_ estimates from 72 h DHA growth inhibition assays (26) were available for 22 isolates (Table S1) but were not considered in the analysis of potential phenotype-genotype associations because this assay does not reflect physiologic exposure to artemisinin.

**TABLE 2:** Summary of mean *in vitro* susceptibility to ART, CQ, and LUM of 40 *P. falciparum* clinical isolates^*a*^

Serial	Culture line ID	Year isolated	Origin	ART (% survival, RSA)	LUM (EC_50_, nM)	CQ (EC_50_, nM)
Comparator lines	*Pf3D7*	–	*Netherlands*	*2.31*	*24.71*	*8.61*
	*Cam3.II^REV^*	–	*Cambodia*	*4.34*	*33.89*	*151.36*
	*Cam3.II^C580Y^*	–	*Cambodia*	*12.21*	–	–
	*Cam3.II^R539T^*	–	*Cambodia*	*31.53*	*28.30*	*198.73*
1	HL1402	2014	Kenya	–	34.72	12.27
2	HL1601	2016	Uganda	6.74	21.57	**101.88**
3	HL1901	2019	Angola	9.39	46.97	**66.72**
4	HL1902	2019	Kenya	9.76	51.26	8.76
5	HL1903	2019	–	–	30.32	**107.80**
6	HL1904	2019	Chad/Sudan	**19.01**	79.93	7.23
7	HL2000^*c*^	2020	Nigeria	3.00	86.77	8.01
8	HL2001^*c*^	2020	Nigeria	3.74	**88.01**	12.26
9	HL2002	2020	Sierra Leone	**21.81**	**96.25**	7.16
10	HL2004	2020	Sudan	3.42	31.61	**74.09**
11	HL2102	2021	Sudan	2.53	46.85	**122.61**
12	HL2103	2021	Zambia	**15.78**	83.71	8.58
13	HL2104	2021	Sierra Leone	7.21	72.50	15.86
14	HL2105	2021	Ghana	7.89	65.00	12.94
15	HL2201^*d*^	2022	Angola	4.77	23.12	11.14
16	HL2202^*d*^	2022	Angola	5.50	31.72	**17.38**
17	HL2203	2022	Nigeria	7.64	29.84	11.69
18	HL2204	2022	Ghana	9.09	30.64	**16.05**
19	HL2205^*b*^	2022	Kenya	9.51	22.63	**94.99**
20	HL2206	2022	Uganda	7.07	34.49	8.35
21	HL2207	2022	Nigeria	**10.67**	**296.41**	14.38
22	HL2208	2022	Uganda	**19.85**	**220.59**	**16.83**
23	HL2209	2022	Nigeria	3.50	**109.90**	**94.19**
24	HL2210^*b*^	2022	Uganda	**13.25**	**225.39**	10.96
25	HL2211	2022	Nigeria	2.13	–	–
26	HL2212	2022	Uganda	7.45	27.36	14.08
27	HL2213^*b*^	2022	Uganda	9.62	50.29	12.18
28	HL2214	2022	Uganda	**10.51**	37.26	15.38
29	HL2301^*b*^	2023	Côte d'Ivoire	5.41	80.05	6.34
30	HL2302	2023	Uganda	7.77	**138.99**	12.85
31	HL2303	2023	Uganda	4.80	**147.53**	10.20
32	HL2304	2023	Zambia	7.18	77.46	9.59
33	HL2305^*b*^	2023	Namibia	**17.99**	86.75	10.53
34	HL2306	2023	South Africa	6.68	74.18	9.81
35	HL2307^*e*^	2023	Sierra Leone	4.46	50.91	10.01
36	HL2308^*e*^	2023	Sierra Leone	4.16	60.18	10.40
37	HL2309	2023	Uganda	5.39	**114.89**	11.09
38	HL2310	2023	Uganda	5.24	**112.57**	13.72
39	HL2401	2024	Uganda	**11.09**	71.25	7.86
40	HL2402	2024	Malawi	**9.77**	58.52	14.88

^*a*^ Denominators are as given in Fig. 1 to 3. –, missing or no data; ART, dihydroartemisinin; CQ, chloroquine; LUM, lumefantrine. Bold indicates mean susceptibility measure at or above top quartile threshold (9.77%, 88.01 nM, 16.05 nM, respectively). Italics indicate comparitor lines (standard controls).

^*b*^ Harbors pfk13 propeller domain variant.

^*c*^ HL2000, HL2001 are independent isolates from one hyperparasitemic individual, taken 2 days apart.

^*d*^ HL2201 and HL2202 are independent isolates from one hyperparasitemic individual, taken 2 days apart.

^*e*^ HL2307 and HL2308 are independent isolates from one individual taken 3 weeks apart, pre/post-AL failure.

**Fig 2:**
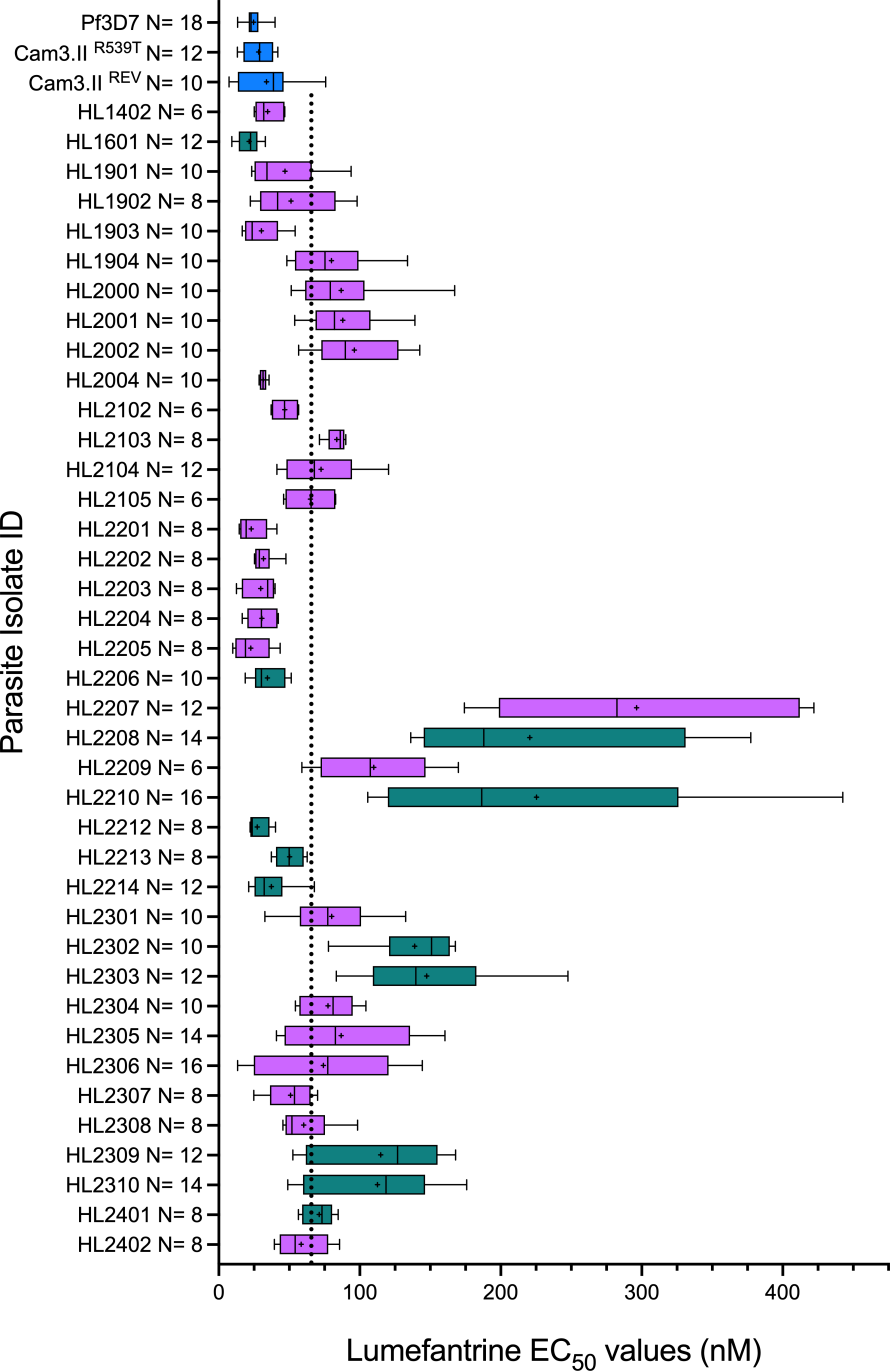
Lumefantrine susceptibility of 39 clinical isolates and three comparator lines. EC_50_ estimates for HL parasite lines and three comparators. Color coding, boxes, and whiskers as in Fig. 1. Denominators for each isolate are shown on y-axis. Vertical dotted line indicates unweighted median EC_50_ estimate among HL lines of 65.0 nM (*N* = 39).

**Fig 3:**
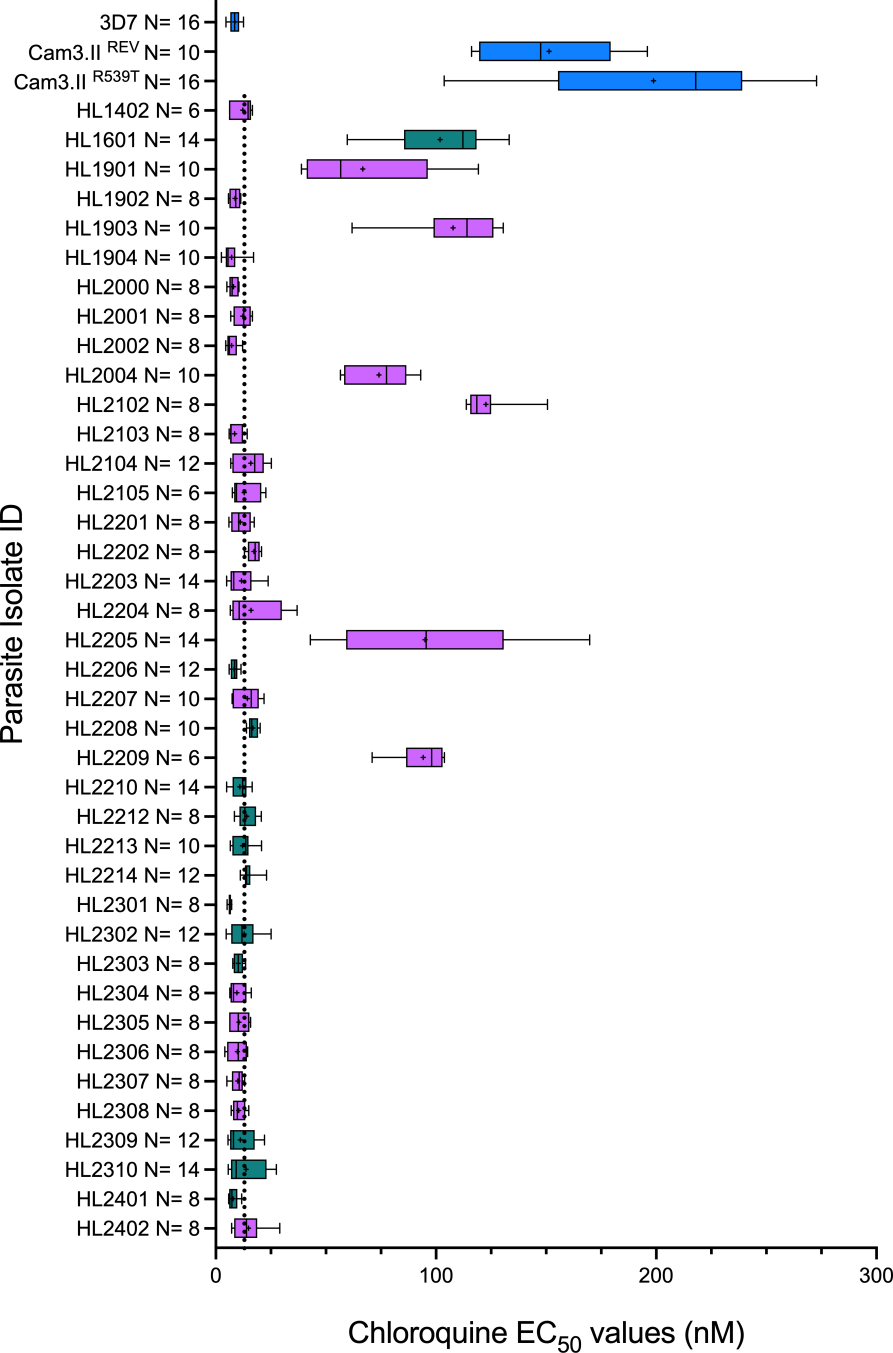
Chloroquine susceptibility of 39 clinical isolates and three comparator lines. EC_50_ estimates for HL parasite lines and four comparators. Color coding, boxes, and whiskers as in Fig. 1. Denominators for each isolate are shown on y-axis. Whiskers represent 95% confidence intervals around the mean of replicates, indicated by **+**. Vertical dotted line indicates unweighted median EC_50_ estimate among HL lines of 12.26 nM (*N* = 39).

Overall mean RSA survival was 8.44% (95% CI 6.82–10.06), and the median was 7.33% (IQR 4.80%–9.77%) (Fig. 1). As these are uncloned parasite lines and are expected to comprise multiple genotypes, variability among replicates for each line is high. In pre-defined comparisons, neither East African origin of the parasite strain (exact *P* = 0.240) nor Ugandan origin in particular (exact *P* = 0.312) was associated with higher survival in the RSA. However, isolates from patients with a history of clinical AL treatment failure featured elevated RSA survival (*N* = 12; median survival 9.58%, IQR 6.96%–13.25%) compared to those from patients who were successfully treated (*N* = 26; median survival 6.88%, IQR 4.77%–10.51%; exact *P* = 0.0487). In particular, six parasite lines isolated from patients that experienced recrudescent malaria after receiving AL treatment in the UK exhibited the greatest RSA survival: HL2002 (Sierra Leone, 21.81%), HL2208 (Uganda, 19.85%) (15), HL2305 (Namibia, 17.99%), HL2210 (Uganda, 13.25%) (15), HL2401 (Uganda, 11.09%) and HL2402 (Malawi, 9.77%). Of these six isolates with the greatest RSA survival, only HL2305 and HL2210 harbored K13 propeller domain mutations, featuring P443S and A675V, respectively.

### Determination of lumefantrine susceptibility in HL-series parasite lines

Next, we examined lumefantrine and chloroquine susceptibility of these isolates using standard 72 h *in vitro* exposure-response assays. Figure 2 presents mean estimates of lumefantrine EC_50_ for 39 parasite lines derived from recent clinical isolates (mean 78.16 nM, 95% CI 58.77–97.56 nM; median 65.00 nM, IQR 34.49–88.01). Figure 3 presents mean estimates of chloroquine EC_50_ for 26 isolates. The distribution of lumefantrine susceptibility was continuous, in contrast to the dichotomous distribution seen for chloroquine, with clear delineation of “sensitive” and “resistant” isolates based on EC_50_ above or below 100 nM (26) (Fig. 3). Median lumefantrine EC_50_ for the 12 isolates of Ugandan origin was 91.91 nM (IQR 35.88–143.26 nM), whereas for the 27 African isolates of non-Ugandan origin the median lumefantrine EC_50_ was 60.18 nM (IQR 31.72–83.71 nM) (exact Wilcoxon *P* = 0.284). Isolates of Ugandan origin (*N* = 12) were more likely to have a lumefantrine EC_50_ in the top quartile (>88 nM) compared to isolates of other African origin (*N* = 27), but this difference was not statistically significant (OR 2.86, 95% CI 0.545–14.8; exact *P* = 0.164). All parasite lines with an elevated EC_50_ estimate for lumefantrine were fully susceptible to chloroquine *in vitro* (Fig. 3).

Several clinical isolates display substantially reduced lumefantrine susceptibility. As previously reported (15), Ugandan isolates HL2208 (exact *P* < 0.001) and HL2210 (exact *P* < 0.001) displayed significantly higher lumefantrine EC_50_ than HL1601, which represents a fully susceptible East African parasite from 2016. Isolates HL2302, HL2303, HL2309, and HL2310 from UK travelers with Ugandan travel history plus HL2207 and HL2209, both of Nigerian origin, also displayed significantly elevated lumefantrine EC_50_ estimates, compared to HL1601 (exact *P* < 0.001 in each case). The reference cell lines 3D7, Cam3.II^R539T^, and Cam3.II^REV^ all display high susceptibility to lumefantrine, as previously observed (15).

Susceptibility estimates from 72 h growth inhibition assays for desethyl amodiaquine, atovaquone, mefloquine, piperaquine, and pyronaridine were available for 22 isolates (Table S1). Although all lines appeared susceptible to desethyl amodiaquine, atovaquone, dihydroartemisinin, mefloquine, piperaquine, and pyronaridine in this assay, there was some evidence that mefloquine was less effective against the 11 lines of Ugandan origin (median EC_50_ 30.78 nM, IQR 26.47–39.02) than against the 11 lines of other origins (median EC_50_ 21.34 nM, IQR 19.27–26.61; Wilcoxon exact *P* = 0.0104).

### Genetic variation in pfk13 propeller and non-propeller domains

**Fig 4:**
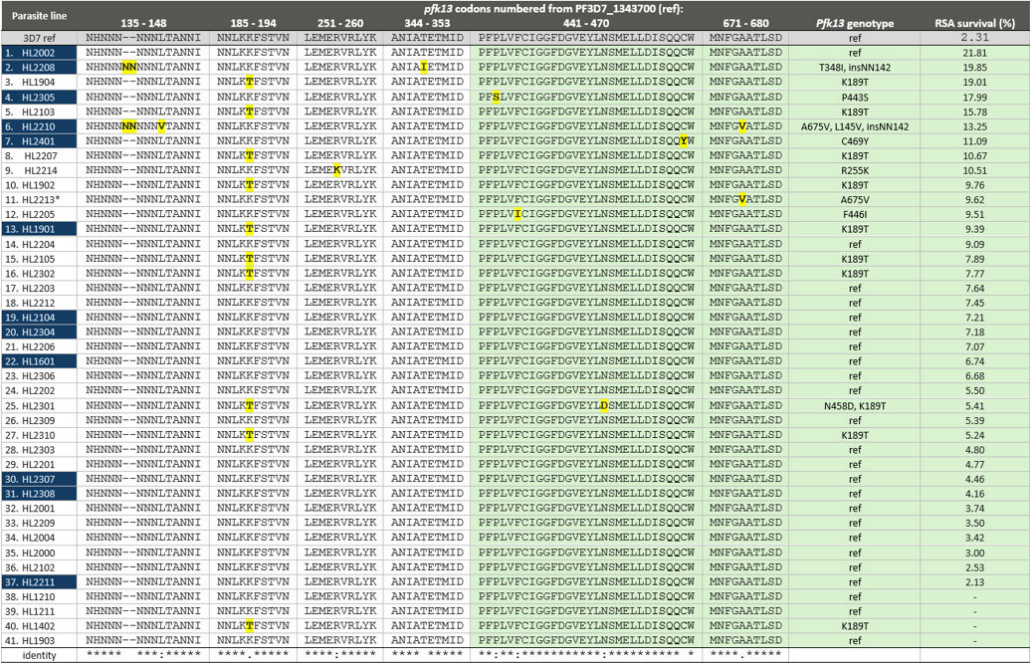
Clustal omega alignment of full-length pfk13 DNA sequences (codons 1–726) from 41 HL-series isolates showing variant positions. The parasite lines are ordered from highest mean RSA survival to lowest. Green shading indicates the kelch propeller domain. Twelve parasite lines, highlighted with dark blue, were isolated from patients reported to have failed treatment with artemether-lumefantrine. Codons not shown are identical to the reference sequence. Data for HL1210 are from WGS analysis only. A dash indicates that RSA data were not available from that parasite line. *HL2213 carries a mixture of both reference and variant residues at codon 675.

A number of non-synonymous polymorphisms in the *pfk13* locus were found by full-length sequencing of cultured parasites from the HL-series (Fig. 4). Of the 41 full-length sequences obtained, 17 encoded amino acid variants at one or more codons. In the propeller domain, the previously described A675V Ugandan variant was seen in HL2210 (6, 15), but also as a mixed genotype with the reference allele in HL2213, which displayed 9.6% survival in the RSA (Table 2). This parasite line had previously been described as having the reference sequence only, but additional sequencing in the current study confirmed the presence of both alleles at this position. Surprisingly, we identified the F446I variant *pfk13* allele, previously only described at the Myanmar-China border (33), in isolate HL2205 from a patient who had traveled to Kenya. Mean RSA survival was 9.5% (*N* = 10), significantly higher than 3D7 (2.3%, *N* = 14; *P* = 0.001). We also identified two novel propeller domain variants: P443S in HL2305, a line of Namibian origin with a markedly elevated mean survival estimate in the RSA of 18.0%, and N458D in HL2301 from Côte d’Ivoire, which featured low RSA survival of 5.4%, comparable to 3D7.

We also found mutations in *pfkelch13* outside of the propeller domain. Strikingly, HL2208, a parasite derived from Uganda, featured the highest mean RSA survival of all tested isolates and carried the previously undescribed upstream variant T348I (incorrectly annotated as T349I by (15). This mutation occurs downstream of a two-asparagine insertion within the 5-Asn tract at positions 137 to 142, recently associated with increased survival in the RSA through gene-editing experiments (34) (Fig. 4). HL2210, derived from a previously described UK malaria patient who failed AL treatment twice (15), also harbors this 2-Asn insertion, as well as the previously undescribed non-propeller variant L143V. These upstream polymorphisms in the HL2210 *pfk13* sequence occur in addition to the A675V propeller domain variant noted above and associated with slow parasite clearance in Ugandan patients under artemisinin treatment (6, 11). HL2210 features elevated RSA survival (13.3%) and markedly elevated lumefantrine EC_50_ (HL2210: 225.4 nM vs 3D7: 24.7 nM). Two further non-propeller domain variant alleles were observed: the previously described and relatively common K189T in 10 parasite lines, and R255K in HL2214, previously reported in a single individual from Senegal (35). Interestingly, HL2214 was among the parasite lines with the top five mean survival estimates in the RSA (Table 2 and Fig. 4), but carries no other polymorphism in *pfk13* (Fig. 4). However, RSA survival was highly variable in this isolate. In six experiments (12 replicates), RSA survival ranged from 1.2% to 29.2%, strongly suggesting genetic heterogeneity among sequential culture population expansions prior to each round of testing. We have previously presented evidence of *in vivo* drug selection on the pfk13 locus of peripheral blood parasites in the AL-treated individual from whom HL2210 was isolated (15). In the current study, HL2000 and HL2001, HL2201 and HL2202, HL2307 and HL2308 represent pairs of isolates taken from the same patient, with the second isolate obtained 1, 2, and 22 days after commencement of treatment, respectively. As all six isolates harbored only the DHA-susceptible reference allele of pfk13, we could not evaluate the impact of treatment on minor alleles.

The six isolates with a non-reference *pfk13* propeller domain genotype were more likely to be higher ranked for relative survival in the RSA than the 31 isolates with the reference (wild type) propeller domain sequence (exact *P* = 0.044). However, if we consider all 16 parasite lines harboring any *pfk13* variant, including K189T, the association with RSA survival is much stronger. These 16 lines have a median relative survival in the RSA of 10.14% (IQR 8.64%–14.52%), whereas the 21 lines with *pfk13* sequence identical to the reference genotype have a median survival of 5.39% (IQR 3.74%–7.18%; exact *P* < 0.001). This suggests that among this group of isolates, non-propeller domain mutations in *pfk13* contribute to observed artemisinin susceptibility phenotypes *in vitro*.

### Genetic variation in the pfubp1 gene

We have previously examined two regions of the *pfubp1* gene encoding 3 amino acids (AA) and 9 AA repeats, respectively, in isolates of *P. falciparum* (14–16). Additional polymorphic UBP1 residues described recently by Casanova et al*.* (29) were assessed in two additional gene fragments. These four gene regions are described in 5′ to 3′ order as fragment I (3 AA repeats, reference codons 1468–1562), fragment II (7 AA repeats, 1893–2339), fragment III (4 AA repeats, 2519–2847), and fragment IV (9 AA repeats, 3064–3160). Although high-quality sequence data were not obtained for all four fragments in each isolate, the available results are presented in Fig. SI through SIV; Table 3, including non-synonymous mutations in *pfubp1* identified by WGS in eight isolates. No evidence of linkage among these regions is apparent, and no stable multi-fragment haplotypes seem to be present among this sample set. This includes the “reference” haplotype: sequence identity to the 3D7 allele occurred at no more than two of the four sequenced regions in any of the evaluable lines. A number of non-synonymous single-nucleotide polymorphisms (SNPs) were identified flanking the repeat regions, including the K2238R variant (29). This allele is present in the reference genome and in six other isolates, although two of these, HL2000 and HL2001, are separate isolates of Nigerian origin from a single hyperparasitemic patient collected on different days. SNPs identified here for the first time include S2281P (HL1211), R2226K (HL2205), and R3113L (HL2208; previously annotated incorrectly as R3105L), plus the double substitutions K1920N/E1921K (HL2301), and G1900R/K2238R (HL2300). Finally, we observed the triple substitution E2193K/L2200I/K2596N (HL2211). The WGS data from eight parasite lines confirm significant sequence diversity in the *pfubp1* locus, with some new variants identified. Variants recently identified by Casanova et al*.,* seen in our data set, include R1133S (HL2104, HL2207, HL2212, HL2213) and the paired substitutions K1914N/E1915K, which occurred together in 7 of the 8 evaluable WGS data sets.

**TABLE 3:** Sequence diversity in the *pfubp1* gene from 39 HL-series isolates^*a*^

Parasite line	*pfubp-1* fragment (codons)				WGS data (≥33.3% read depth)
	I (1468–1562)	II (1893–2339)	III (2519–2847)	IV (3064–3160)	
3D7_ref	2-2-1-0-1-1	1-3-2-2-1 K2238R^*b*^	2-1-1-1	2-1-0-0-4	
HL1211	ref	2-2-2-2-1 S2281P	2-1-1-2	2-1-0-0-3	
HL1402	ref			ref	
HL1601	2-1-2-0-1-1	2-2-2-2-1	2-1-1-2	2-1-0-0-3	
HL1901			2-1-1-2		
HL1902	ref	2-2-3-2-1	2-0-2-1		
HL1903	2-3-0-0-1-1	2-2-2-2-1	2-1-1-2		
HL1904	2-2-0-0-1-1	2-2-3-2-1	ref		
HL2000**^*c*^**	ref	2-2-2-2-1 K2238R	ref		
HL2001**^*c*^**	ref	2-2-2-2-1 K2238R	ref		
HL2002	2-3-0-0-1-1	2-2-3-2-1	2-1-2-1		
HL2004	ref	2-2-2-3-1	2-1-2-1		
HL2102	ref	2-2-2-3-1	2-1-2-1	2-1-0-0-2	
HL2103	2-3-2-0-1-1	2-2-2-2-1	2-1-2-0		
HL2104	2-1-1-0-1-1	2-2-2-2-1 K2238R	ref	2-1-0-0-3	Y615H N759K N789D D795N R1133S K1914N E1915K S3103N
HL2105	2-2-1-1-1-1 E1528D	2-2-2-2-1	ref		
HL2201**^*c*^**	ref	2-2-1-3-1	2-1-2-1 H2663C/S2635C		
HL2202**^*c*^**	ref	2-2-1-3-1	2-1-2-1 H2663C/S2635C	2-1-0-1-3	
HL2203	ref	2-2-2-2-1	2-2-1-2	2-1-0-0-3	
HL2204	ref	2-2-2-2-1	ref	2-1-0-0-3	
HL2205	ref	2-2-2-3-1 R2226K	2-1-1-2	ref	K447E Y615H N789D N798D S956N N1710S K1914N E1915K R2226K K2238K
HL2206	ref	2-2-2-3-1	2-1-1-2	2-1-0-0-3	
HL2207					D606N Y615H N789D D795N R1133S K2238K D2704E
HL2208	ref	3-1-2-2-1	ref	2-1-0-0-3 R3113L	
HL2209	ref	2-2-2-2-1	2-2-1-2	2-1-0-1-2	
HL2210	2-3-1-0-0-1 Y1530C	2-2-2-2-1	ref	ref	N562D Y615H N784D K785D Y1530C N1891K K1914N E1915K R2238K
HL2211	2-1-1-0-1-1	2-2-3-2-1 E2193K/L2200I	2-1-3-1 K2596N		
HL2212	ref	2-2-3-2-1	ref	1-0-1-0-2	Y615H D777G N789D D791N N931D R1133S K1914N E1915K K1928N E1929K R2238K H3083R N3085S N3094S
HL2213	ref	2-2-2-2-1	ref	2-1-0-0-3	D41G N101I Y615H D795N N931D R1133S T1294K K1914N E1915K
HL2214	2-2-0-0-1-1	2-2-4-2-1 E1921K	ref		
HL2301	2-1-1-0-1-1	2-3-2-2-1 K1920N/E1921K	2-1-2-1		
HL2302	ref	3-2-2-2-1	1-1-1-2		
HL2303	2-2-0-0-1-1	3-2-2-2-1 G1900R/K2238R	2-2-1-2		
HL2304	ref	2-2-2-3-2	2-1-1-2		
HL2305	2-2-2-0-1-1	2-2-3-1-2 E1921K	2-1-1-2		Y615H V708I N754K K774N D777G K779N D1289E N1710S K1914N E1915K E1929K K1949N K1956N K2238K C2818Y D3088N G3089RD V3090A
HL2306	2-3-0-0-1-1	3-2-2-2-1	2-2-1-1		
HL2307**^*c*^**	2-3-1-1-1-1 E1528D	2-2-2-2-1 K2238R	2-2-1-2		D267E D606N Y615H K764N K774N D777GN789D D795N E1528D K1914N E1915K C2818Y
HL2308**^*c*^**	2-3-0-0-1-1	2-2-2-2-1 K2238R	2-2-1-2		
HL2309	ref	2-2-3-1-1	2-1-1-2		
HL2310	2-2-0-0-1-1				

^*a*^ For each of the four repeat regions analyzed by direct PCR sequencing, array codes are based on alignments (Fig. S1 through S4). Non-synonymous SNPs within the four fragments are shown. The final column lists variants identified by WGS, which was available for eight isolates. Clone multiplicity means many of these were present in addition to the reference residue. We set an arbitrary prevalence threshold of 33% of read depth to include a variant here. “ref” denotes 100% identity with the reference allele. Light gray fill denotes data unavailable.

^*b*^ Wild-type residue at codon 2238 is Lys, reference genome encodes the Arg variant.

^*c*^ HL2000/HL2001, and HL2201/HL2202, are pairs of isolates from two hyperparasitaemic individuals (Nigeria 2020 and Angola 2022, respectively) taken 1–2 days apart. HL2307 and HL2308 are also from a single individual, the latter taken 3 weeks later following post-treatment recrudescence.

Previous studies implicated the *pfubp1* variant E1528D in artemisinin susceptibility (16, 36). This variant occurred in two isolates, HL2105 and HL2307, neither of which displays elevated survival in the RSA. The two Ugandan lines HL2208 and HL2210 harbor unique *pfubp1* substitutions, R3113L and Y1530C, respectively, and both display markedly reduced susceptibility to lumefantrine (Fig. 2) (15). We have no evidence that either of these *pfubp1* alleles contributes to this phenotype, but this can be explored in future studies using gene editing approaches. *Pfubp1* sequence variants I1787N and P3264T, orthologues of *P. yoelii* mutations implicated in lumefantrine resistance (23), were not seen among our sequenced isolates.

### *Polymorphisms in* pfap2μ, pfcoronin, pfcrt, *and* pfmdr1

Full-length *pfap2µ* sequence data were obtained for 25 isolates. In addition, WGS data were available for 10 of these and used to confirm the polymorphisms observed. Inserts of a single residue in poly-asparagine tracts at codons 227–233 and/or 320–324 were seen in seven of the sequenced HL lines. The S160N variant was present in HL2208 and HL2305, whereas HL2304 harbored two variants: S160N and the previously unreported K199T. Finally, the Ugandan isolate HL2213 harbored two variants described here for the first time: N233K and F437L. Although S160N has been previously implicated in drug susceptibility (16, 37), when introduced into the 3D7 laboratory strain, there was no impact on *in vitro* artemisinin susceptibility, in contrast to the marked impact of the I592T variant, which has not been found in natural isolates (21). Nevertheless, all three isolates carrying *pfap2μ* S160N were from individuals who failed treatment with AL. The novel *pfap2mu* variants described here may also be of interest but require further validation *in vitro* and *in vivo* before any relevance to drug susceptibility can be ascribed to them.

Codons 43 to 190 of *pfcoronin* were amplified and sequenced for 25 parasite lines (Table S2). HL2208 and HL2301 both harbored the P76S variant, which has been reported from several African countries. Much more common was S183G, present in 14 of 21 sequences obtained and distributed widely; this allele was present in 3 of 7 West African, 6 of 9 East African, and all five Southern African sequences. All other isolates and codons within this directly sequenced region were identical to the 3D7 reference. Full-length WGS sequence data on *pfcoronin* were available for 12 lines and identified one ubiquitous synonymous change (A→G, K212K) and two additional SNPs encoding amino acid changes downstream of our direct-sequenced gene fragment: V424I in HL1210, HL2207, and HL2307 (all West African), and F434L present in HL1210 alone.

Of 26 evaluable lines, 6 (23.1%) harbored the chloroquine resistance-associated *pfcrt* genotype CVIET at codons 72–76, in each case corresponding to elevated chloroquine EC_50_ (Fig. 3). All 25 parasite lines that were successfully tested harbored only a single copy of the *pfmdr1* locus; 1 of 26 (HL1210, Ghana) harbored the N86Y variant and 13 of 26 the Y184F variant, known to be selected by AL use (38). Thus, the aminoquinoline-susceptible *pfmdr1* haplotypes NYSND and NFSND at codons 86, 184, 1034, 1042, and 1246 were predominant among the lines evaluated, as previously observed in post-ACT African settings (16, 39). Among the 12 lines for which WGS data were obtained, additional variants of *pfcrt* identified included A220S (2), Q271E (2), I356T (1), and R371I (3), whereas in *pfmdr1,* two isolates carried the dual substitutions D650N and N652D (Table S2).

**Fig 5:**
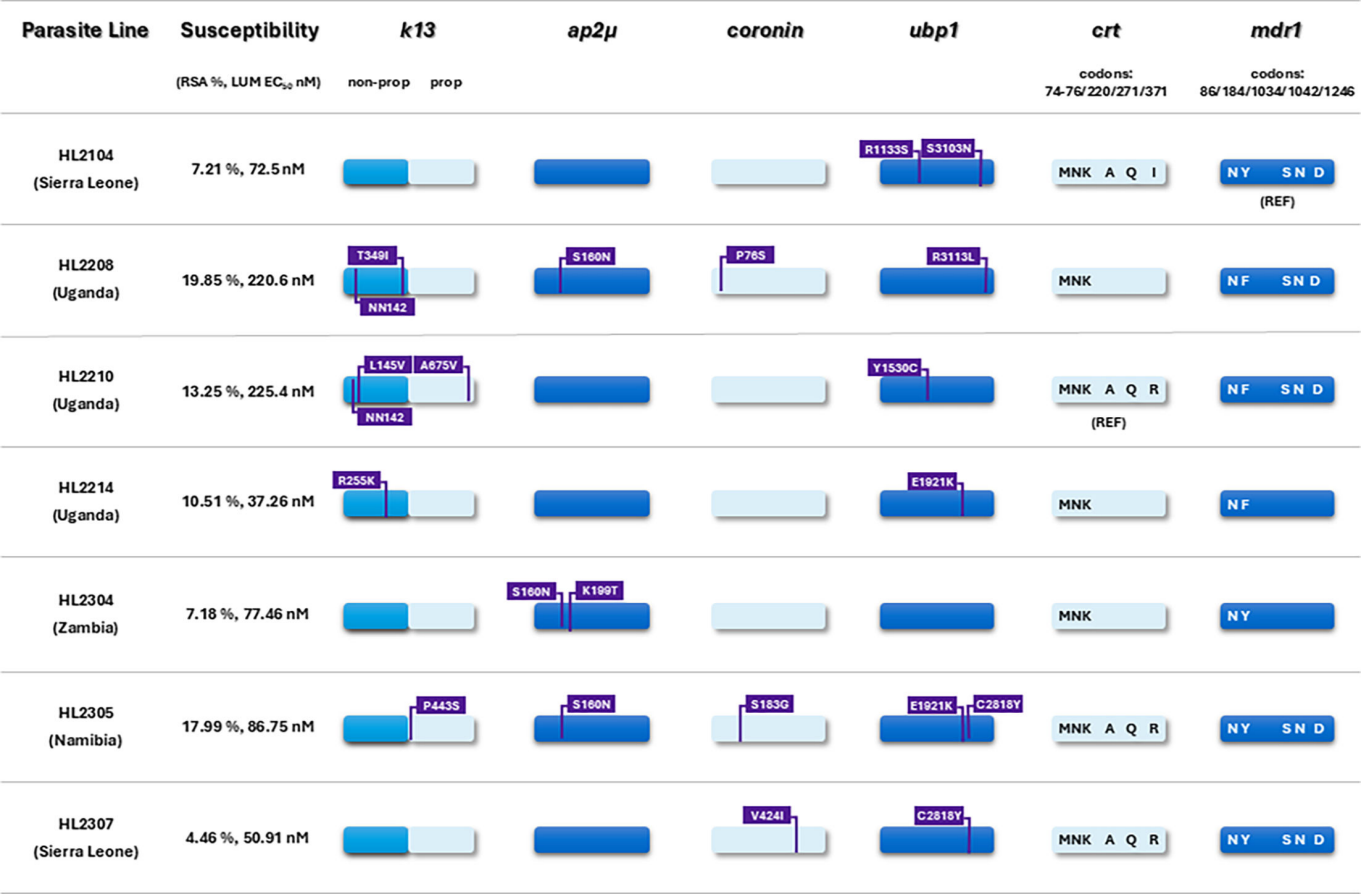
Combined multi-locus haplotypes for *pfk13, pfap2m*, *pfubp1*, *pfcoronin, pfcrt,* and *pfmdr1* genotypes for seven isolates from patients who failed AL treatment. Mean survival in the RSA and mean lumefantrine EC_50_ are shown for each of these lines. For four of these genes, non-synonymous variants of interest are shown together with *pfcrt* and *pfmdr1* haplotypes at the codons shown. WGS data were not available for HL2208, HL2214, and HL2304, and these isolates were evaluated by PCR genotyping only, so they are missing information at some positions.

To explore the importance of multi-locus genotypes further, we collated data for the six loci *pfk13*, *pfap2μ*, *pfubp1*, *pfcoronin*, *pfcrt,* and *pfmdr1* for seven parasite lines from patients with AL treatment failure, each of which had all six gene sequences available (Fig. 5). Although descriptive only, the analysis indicates that the two lines with the highest RSA survival, HL2208 and HL2305, both carry non-synonymous substitutions in four of the genes: *pfk13*, *pfap2μ*, *pfubp1,* and *pfcoronin*. This suggests that variants of the latter three loci may have an additive effect on *pfk13* -mediated reduced DHA susceptibility.

## DISCUSSION

In this study, we have assessed *in vivo* treatment outcomes, artemisinin and lumefantrine *in vitro* susceptibility phenotypes, and gene sequences for resistance-associated loci for 50 *P. falciparum* parasite lines of African origin isolated from UK travelers between 2012 and 2023. Among this collection of *in vitro* cultured lines were 11 from patients who had traveled to Uganda. Six of these lines demonstrated significantly reduced lumefantrine susceptibility *in vitro*. All remaining lines tested displayed effective growth inhibition by lumefantrine, a key component of AL, which is the most commonly used ACT. Artemisinin susceptibility was also compromised in several lines from different African countries, with the eight lines in the top quartile presenting with mean RSA survival estimates of 9.42% to 19.85% and originating from Uganda (*n* = 4), Namibia, Zambia, Nigeria, and Kenya (each *n* = 1). Given the very large numbers of *P. falciparum* cases presenting in the UK over this 12-year period and the small number of treatment failures encountered, we do not find evidence of a serious resistance threat at present to AL effectiveness in returning travelers. However, some of the parasite lines we have established do display phenotypes of concern and can provide an opportunity to identify genetic markers and biological mechanisms of emerging *P. falciparum* resistance to both component drugs in the AL combination. No evidence was found among our isolates of a threat to the efficacy of other ACT partner drugs, including amodiaquine, pyronaridine, and piperaquine. Meanwhile, our findings have prompted updated guidance from the UK Malaria Expert Advisory Group on the management of treatment failure (recrudescence) in falciparum malaria, by advocating the inclusion of a second ACT, dihydroartemisinin-piperaquine, in UK treatment guidelines (40).

Partial resistance to artemisinin is increasingly recognized as a serious public health concern in the eastern countries of Africa. Among the eight parasite lines with high survival in the RSA, only four carried previously recognized and validated genetic markers of partial artemisinin resistance in the propeller domain of the *pfk13* locus: two Ugandan isolates carried the well-recognized A675V allele, one Ugandan isolate carried C469Y, and a Kenyan isolate carried F446I. This latter finding was unexpected, as this mutation emerged on the Myanmar-China frontier a decade or more ago and has not previously been reported in Africa, to our knowledge (33, 41, 42). Novel mutations encoding amino acid substitutions in *pfk13* were, in the propeller domain, P443S (Namibia) and N458D (Côte d’Ivoire), and in the non-propeller domain L143V (Uganda) and R255K (Uganda). We also observed higher survival in the RSA among seven lines carrying only the *pfk13* K189T non-propeller variant (Fig. 4). Mutations of interest were also found among these lines in other loci implicated in artemisinin susceptibility: *pfubp1*, *pfap2μ,* and *pfcoronin,* and there was some evidence of a possible additive effect of these multi-locus genotypes on RSA survival in the case of HL2208 and HL2305 (Fig. 5)*.* The only two parasite lines exhibiting all three phenotypes of concern—AL treatment failure *in vivo* and reduced susceptibility to both lumefantrine and artemisinin *in vitro*—were HL2208 and HL2210, as previously described (15).

Our findings suggest that artemisinin susceptibility may be compromised by a variety of mechanisms that can arise independently at different geographic locations. These lead to slow clearance *in vivo* and enhanced artemisinin survival *in vitro* as evidence of partial resistance, but to date, there is no evidence of a powerful directional selection for one or two dominant genotypes that are rapidly spreading beyond their region of origin. Nevertheless, cross-border movement of several East African *pfk13* alleles has occurred, and a more serious and swift-moving threat may yet become evident (43). Although *pfk13-*associated mechanisms are clearly very important, the finding of high RSA survival in the absence of *pfk13* mutations in isolates HL2002 and HL2204 indicates a need for vigilance in seeking to identify polymorphisms that may underlie K13-independent reduced artemisinin susceptibility in African *P. falciparum* populations. This suggests that a full understanding of the partial artemisinin phenotype requires careful consideration of multigenic haplotypes encompassing *pfk13* together with the loci set out in Fig. 5 and others identified by future studies.

Treatment failure in malaria patients does not always indicate parasite adaptations leading to partial or complete drug resistance. *P. falciparum* recrudescence of an infection treated with AL can be the result of poor drug absorption, due to variability in liver metabolism among individuals, leading to inadequate activation of pro-drugs and underdosing, particularly in subjects with higher body-mass index (13). We cannot rule out these factors as major contributors to treatment failure among the patients in this study. However, for several patients with documented recrudescence of *P. falciparum*, parasite factors are important in the observed drug failure *in vivo*, given the presence of validated markers of partial artemisinin resistance (such as *pfk13* A675V) or significantly reduced growth inhibition *in vitro* in these studies. However, we lack validated genetic markers or an understanding of resistance mechanisms for the lumefantrine component of AL, and rectifying this must now be a high priority for current work, which aims to preserve the effectiveness of treatment for falciparum malaria in Africa.

Our study provides a number of potentially useful findings, but also suffers from some limitations. Over the time period during which the 50 *P. falciparum* isolates were established in *in vitro* culture, the criteria for selecting an isolate for *in vitro* parasite adaptation changed according to the availability of lab resources, staff priorities, and research objectives at the time of sampling. This has a clear impact on the generalizability of our findings, which cannot be considered formally representative of the biological characteristics of *P. falciparum* infections imported into the UK, nor of parasite populations in the countries of origin. A second limitation is that for many of the isolates, particularly those adapted between 2013 and 2018, we do not have complete data sets available. However, each of these lines has been stably cryo-preserved, and future work to fill some of these gaps is possible in the future. This may be particularly important if, in the countries from which these understudied parasite lines originated, there is a future emergence of new resistance phenotypes so that comparison with our earlier HL lines from the same area would be instructive. Thirdly, as with all studies of imported malaria, geographic origin cannot be specified below the country level except in a small minority of cases, and even this is dependent on patient recall and the fidelity of communication to the UK MRL from the primary health facility or hospital where the patient was diagnosed and treated. The work described herein is focused on patient needs and performed under routine care conditions in the UK National Health Service. We therefore do not benefit from the rigor of documentation implemented by prospective research studies, and so no attempt was made to interpret findings at greater granularity than is appropriate with the routine data we receive.

An additional concern with our data set is that the lumefantrine EC_50_ estimates reported are higher than those generally reported in the literature, including in recent studies of Ugandan-origin parasites (8). The most likely explanation for this is that our laboratory performs susceptibility testing in medium that includes human serum, known to bind lumefantrine and therefore potentially reduce the effective exposure of the parasites to the drug compared to the serum-free medium, which is widely used by other groups. In addition, as in *ex vivo* studies, our lines are not cloned and thus have multiple genotypes present, leading to variability in EC_50_ estimates between experiments. Nevertheless, our results show an excellent agreement between EC_50_ estimates for lumefantrine in the cases where we have two isolates from the same patient, established on different days. This suggests our methodology is relatively stable and consistent, and that we have internal validity in our experimental data set. To overcome this issue of inter-lab variation, our better-characterized lines, such as HL2210, can be provided to other laboratories as a “benchmark” for reduced lumefantrine susceptibility, just as the Cam3.II lines developed in the Fidock laboratory have proved useful benchmark lines for artemisinin susceptibility (Table 2 [44]).

Overall, our study provides descriptive phenotypic and genotypic analyses of a collection of African-origin *P. falciparum* lines, including a small number of recent Ugandan isolates with reduced susceptibility to lumefantrine. These lines could be used in genetic crosses with fully susceptible parasites to identify genetic correlates of this phenotype, as has recently been achieved with piperaquine resistance (45), with the aim of validating genetic markers for lumefantrine susceptibility in Africa.

## Data Availability

Raw reads have been deposited in the European Nucleotide Archive (Project Accession: PRJEB94354).

## SUPPLEMENTAL MATERIAL

Supplemental Tables mbio.03676-25-s0001.docx Tables S1 and S2.

Supplemental Figures mbio.03676-25-s0002.docx Fig. S1 to S4.

ASM does not own the copyrights to Supplemental Material that may be linked to, or accessed through, an article. The authors have granted ASM a non-exclusive, world-wide license to publish the Supplemental Material files. Please contact the corresponding author directly for reuse.
