# Supplementary material for: Novel Pfk13 and Pfubp1 genotypes in African Plasmodium falciparum isolates exhibiting reduced susceptibility to the antimalarials artemisinin and lumefantrine
Source: mBio. 2026 Feb 25;17(4):e03676-25. doi: 10.1128/mbio.03676-25 (PMC13059730; doi:10.1128/mbio.03676-25)
Supplement: Supplemental Tables — Tables S1 and S2. [file mbio.03676-25-s0001.docx]

| ***parasite line*** | Year | Geographic origin | Treatment failure | desethyl AQ | atovaquone | dihydroartemisinin | mefloquine | piperaquine | pyronaridine |
| --- | --- | --- | --- | --- | --- | --- | --- | --- | --- |
| 3D7 (ref) | 1987 | Netherlands | 0 | 15.89 | 0.44 | 3.08 | 20.13 | 12.86 | 1.68 |
| HL1601 | 2016 | Uganda | 1 | 26.01 | 0.19 | 2.85 | 27.02 | 19.38 | 1.16 |
| HL2201 | 2022 | Angola | 0 | 9.45 | 0.14 | 3.25 | 22.72 | 16.64 | 2.61 |
| HL2202 | 2022 | Angola | 0 | 10.29 | 0.17 | 4.89 | 37.97 | 17.25 | 3.89 |
| HL2203 | 2022 | Nigeria | 0 |  |  | 3.24 | 31.07 | 15.64 | 2.69 |
| HL2204 | 2022 | Ghana | 0 |  |  | 3.39 | 26.61 | 20.36 | 2.95 |
| HL2205 | 2022 | Kenya | 0 | 26.48 | 0.12 | 1.81 | 21.34 | 16.36 | 2.37 |
| HL2206 | 2022 | Uganda | 0 | 12.57 | 0.12 | 2.67 | 19.95 | 17.68 | 2.65 |
| HL2208 | 2022 | Uganda | 1 | 20.59 | 0.21 | 4.13 | 33.93 | 29.12 | 2.10 |
| HL2210 | 2022 | Uganda | 1 | 14.35 | 0.17 | 3.98 | 39.02 | 19.85 | 1.98 |
| HL2212 | 2022 | Uganda | 0 | 18.48 | 0.17 | 1.73 | 30.78 | 22.04 | 1.85 |
| HL2213 | 2022 | Uganda | 0 | 16.97 | 0.17 | 3.87 | 47.24 | 22.94 | 1.97 |
| HL2214 | 2022 | Uganda | 0 | 13.43 | 0.19 | 1.83 | 29.57 | 20.62 | 2.25 |
| HL2301 | 2023 | Côte d'Ivoire | 0 | 16.97 | 0.23 | 2.65 | 21.70 | 13.54 | 2.28 |
| HL2302 | 2023 | Uganda | 0 | 17.27 | 0.37 | 3.68 | 39.74 | 22.18 | 2.73 |
| HL2303 | 2023 | Uganda | 1 | 21.74 | 0.20 | 2.85 | 33.27 | 15.70 | 3.30 |
| HL2304 | 2023 | Zambia | 1 | 18.82 | 0.17 | 3.68 | 21.21 | 13.34 | 2.68 |
| HL2305 | 2023 | Namibia | 1 | 16.60 | 0.29 | 1.34 | 18.64 | 14.59 | 1.95 |
| HL2306 | 2023 | South Africa | 0 | 17.13 | 0.23 | 2.00 | 17.51 | 13.70 | 1.83 |
| HL2307 | 2023 | Sierra Leone | 1 | 15.11 | 0.08 | 1.71 | 20.50 | 15.57 | 2.23 |
| HL2308 | 2023 | Sierra Leone | 1 | 15.26 | 0.11 | 1.87 | 19.27 | 16.47 | 2.88 |
| HL2309 | 2023 | Uganda | 0 | 17.23 | 0.31 | 3.10 | 26.47 | 20.84 | 3.20 |
| HL2310 | 2023 | Uganda | 0 | 12.03 | 0.12 | 1.77 | 25.78 | 17.73 | 0.98 |

**Supplementary Table 1. Susceptibility of 22 HL lines to desethyl amodiaquine (AQ), atovaquone, dihydroartemisinin, mefloquine, piperaquine and pyronaridine.**

Estimates shown represent the mean EC_50_ in nM of at least 8 biological and technical replicate data points.

| ***parasite line*** | Origin | *pfap2μ*  PF3D7_1218300 | *pfcoronin*  PF3D7_1251200 | *pfcrt*  PF3D7_0709000 | *pfmdr1*  PF3D7_0523000 | *pfdhfr-ts*  PF3D7_0417200 | *pfppk-dhps* PF3D7_0810800 |
| --- | --- | --- | --- | --- | --- | --- | --- |
| HL1210 | Ghana | K331A; T332K | S183G; V424I; P434L | M74I; N75E; K76T; A220S; Q271E; R371I | N86Y; Y184F; D650N; N652D | C59R; S108N | A437G |
| HL2104 | Sierra Leone | ref | ref | R371I | ref | N51I; C59R; S108N | A437G; K540E |
| HL2203 | Nigeria | ref | ref | ref | ref | S108N | A437G; K540E |
| HL2205 | Kenya | ref | S183G | M74I; N75E; K76T; A220S; Q271E; I356T R371I | Y184F | N51I; C59R; S108N | A437G; K540E* |
| HL2206 | Uganda | ref | ref | ref | ref | N51I; C59R; S108N | A437G; K540E |
| HL2207 | Nigeria | ref | S183G; V424I | ref | Y184F | N51I; C59R; S108N | I431V; S436A; A437G; A581G; A613S |
| HL2210 | Uganda | ref | ref | ref | Y184F | N51I; C59R; S108N | A437G; K540E |
| HL2212 | Uganda | ref | S183G | ref | ref | N51I; C59R; S108N; I164L | A437G; K540E |
| HL2213 | Uganda | N233K | S183G | ref | Y184F | C59R; S108N; I164L | A437G; K540E |
| HL2301 | Côte d'Ivoire | ref | P76S | ref | D650N; N652D | N51I; C59R; S108N | A437G; K540E |
| HL2305 | Namibia | S160N | S183G | ref | ref | N51I; C59R; S108N | A437G; K540E |
| HL2307 | Sierra Leone | ref | V424I | ref | ref | N51I; C59R; S108N | A437G; K540E |

**Supplementary Table 2. Genotypes for *pfap2μ, pfcoronin*, *pfcrt, pfmdr1, pfdhfr* and *pfdhps* for 12 parasite lines by WGS analysis.**

Illumina short-read sequencing and mapping of data to the reference genome was carried out as described in Methods. Variant calling was performed using the *Malaria Profiler* web tool (Phelan *et al.*, 2023). Only non-synonymous variants with at least 33% prevalence among reads are presented. Other variants at lower prevalence were seen for some isolates at some loci, as expected for multi-clonal infections. Indels in these loci were noted among these isolates, particularly in low complexity sequence tracts, but are not included in the table.

* K540E was a only minor variant in HL2205 (17% of reads)
