# Supplementary material for: Novel Pfk13 and Pfubp1 genotypes in African Plasmodium falciparum isolates exhibiting reduced susceptibility to the antimalarials artemisinin and lumefantrine
Source: mBio. 2026 Feb 25;17(4):e03676-25. doi: 10.1128/mbio.03676-25 (PMC13059730; doi:10.1128/mbio.03676-25)
Supplement: Supplemental Figures — Fig. S1 to S4. [file mbio.03676-25-s0002.docx]

3D7_ref KDKENKLNMDDDINMNKGNDQDVNRTYKNEKNKEEDKYGKNEKNEKYDKYD---KYE------KYEKYDKYKKDNKNQHDDPLY

HL1211 KDKENKLNMDDDINMNKGNDQDVNRTYKNEKNKEEDKYGKNEKNEKYDKYD---KYE------KYEKYDKYKKDNKNQHDDPLY

HL1402 KDKENKLNMDDDINMNKGNDQDVNRTYKNEKNKEEDKYGKNEKNEKYDKYD---KYE------KYEKYDKYKKDNKNQHDDPLY

HL1601 KDKENKLNMDDDINMNKGNDQDVNRTYKNEKNKEEDKYGKNEKNEKYD------KYEKYE---KYEKYDKYKKDNKNQHDDPLY

HL1902 KDKENKLNMDDDINMNKGNDQDVNRTYKNEKNKEEDKYGKNEKNEKYDKYD---KYE------KYEKYDKYKKDNKNQHDDPLY

HL1903 KDKENKLNMDDDINMNKGNDQDVNRTYKNEKNKEEDKYGKNEKNEKYDKYDKYD---------KYEKYDKYKKDNKNQHDDPLY

HL1904 KDKENKLNMDDDINMNKGNDQDVNRTYKNEKNKEEDKYGKNEKNEKYDKYD------------KYEKYDKYKKDNKNQHDDPLY

HL2000 KDKENKLNMDDDINMNKGNDQDVNRTYKNEKNKEEDKYGKNEKNEKYDKYD---KYE------KYEKYDKYKKDNKNQHDDPLY

HL2001 KDKENKLNMDDDINMNKGNDQDVNRTYKNEKNKEEDKYGKNEKNEKYDKYD---KYE------KYEKYDKYKKDNKNQHDDPLY

HL2002 KDKENKLNMDDDINMNKGNDQDVNRTYKNEKNKEEDKYGKNEKNEKYDKYDKYD---------KYEKYDKYKKDNKNQHDDPLY

HL2004 KDKENKLNMDDDINMNKGNDQDVNRTYKNEKNKEEDKYGKNEKNEKYDKYD---KYE------KYEKYDKYKKDNKNQHDDPLY

HL2102 KDKENKLNMDDDINMNKGNDQDVNRTYKNEKNKEEDKYGKNEKNEKYDKYD---KYE------KYEKYDKYKKDNKNQHDDPLY

HL2103 KDKENKLNMDDDINMNKGNDQDVNRTYKNEKNKEEDKYGKNEKNEKYDKYDKYDKYEKYE---KYEKYDKYKKDNKNQHDDPLY

HL2104 KDKENKLNMDDDINMNKGNDQDVNRTYKNEKNKEEDKYGKNEKNEKYD------KYE------KYEKYDKYKKDNKNQHDDPLY

HL2105 KDKENKLNMDDDINMNKGNDQDVNRTYKNEKNKEEDKYGKNEKNEKYDKYD---KYE---KY**D**KYEKYDKYKKDNKNQHDDPLY

HL2201 KDKENKLNMDDDINMNKGNDQDVNRTYKNEKNKEEDKYGKNEKNEKYDKYD---KYE------KYEKYDKYKKDNKNQHDDPLY

HL2202 KDKENKLNMDDDINMNKGNDQDVNRTYKNEKNKEEDKYGKNEKNEKYDKYD---KYE------KYEKYDKYKKDNKNQHDDPLY

HL2203 KDKENKLNMDDDINMNKGNDQDVNRTYKNEKNKEEDKYGKNEKNEKYDKYD---KYE------KYEKYDKYKKDNKNQHDDPLY

HL2204 KDKENKLNMDDDINMNKGNDQDVNRTYKNEKNKEEDKYGKNEKNEKYDKYD---KYE------KYEKYDKYKKDNKNQHDDPLY

HL2205 KDKENKLNMDDDINMNKGNDQDVNRTYKNEKNKEEDKYGKNEKNEKYDKYD---KYE------KYEKYDKYKKDNKNQHDDPLY

HL2206 KDKENKLNMDDDINMNKGNDQDVNRTYKNEKNKEEDKYGKNEKNEKYDKYD---KYE------KYEKYDKYKKDNKNQHDDPLY

HL2208 KDKENKLNMDDDINMNKGNDQDVNRTYKNEKNKEEDKYGKNEKNEKYDKYD---KYE------KYEKYDKYKKDNKNQHDDPLY

HL2209 KDKENKLNMDDDINMNKGNDQDVNRTYKNEKNKEEDKYGKNEKNEKYDKYD---KYE------KYEKYDKYKKDNKNQHDDPLY

HL2210 KDKENKLNMDDDINMNKGNDQDVNRTYKNEKNKEEDKYGKNEKNEKYDKYDKYDKYE------K**C**EKYDKYKKDNKNQHDDPLY

HL2211 KDKENKLNMDDDINMNKGNDQDVNRTYKNEKNKEEDKYGKNEKNEKYD------KYE------KYEKYDKYKKDNKNQHDDPLY

HL2212 KDKENKLNMDDDINMNKGNDQDVNRTYKNEKNKEEDKYGKNEKNEKYDKYD---KYE------KYEKYDKYKKDNKNQHDDPLY

HL2213 KDKENKLNMDDDINMNKGNDQDVNRTYKNEKNKEEDKYGKNEKNEKYDKYD---KYE------KYEKYDKYKKDNKNQHDDPLY

HL2214 KDKENKLNMDDDINMNKGNDQDVNRTYKNEKNKEEDKYGKNEKNEKYDKYD------------KYEKYDKYKKDNKNQHDDPLY

HL2301 KDKENKLNMDDDINMNKGNDQDVNRTYKNEKNKEEDKYGKNEKNEKYD------KYE------KYEKYDKYKKDNKNQHDDPLY

HL2302 KDKENKLNMDDDINMNKGNDQDVNRTYKNEKNKEEDKYGKNEKNEKYDKYD---KYE------KYEKYDKYKKDNKNKHDDPLY

HL2303 KDKENKLNMDDDINMNKGNDQDVNRTYKNEKNKEEDKYGKNEKNEKYDKYD------------KYEKYDKYKKDNKNQHDDPLY

HL2304 KDKENKLNMDDDINMNKGNDQDVNRTYKNEKNKEEDKYGKNEKNEKYDKYD---KYE------KYEKYDKYKKDNKNQHDDPLY

HL2305 KDKENKLNMDDDINMNKGNDQDVNRTYKNEKNKEEDKYGKNEKNEKYDKYD---KYE------KYEKYDKYKKDNKNQHDDPLY

HL2306 KDKENKLNMDDDINMNKGNDQDVNRTYKNEKNKEEDKYGKNEKNEKYD------KYE------KYEKYDKYKKDNKNQHDDPLY

HL2307 KDKENKLNMDDDINMNKGNDQDVNRTYKNEKNKEEDKYGKNEKNEKYDKYDKYD------KY**D**KYEKYDKYKKDNKNQHDDPLY

HL2308 KDKENKLNMDDDINMNKGNDQDVNRTYKNEKNKEEDKYGKNEKNEKYDKYDKYDKYE------KYEKYDKYKKDNKNQHDDPLY

HL2309 KDKENKLNMDDDINMNKGNDQDVNRTYKNEKNKEEDKYGKNEKNEKYDKYD------------KYEKYDKYKKDNKNQHDDPLY

HL2310 KDKENKLNMDDDINMNKGNDQDVNRTYKNEKNKEEDKYGKNEKNEKYDKYD------------KYEKYDKYKKDNKNQHDDPLY

************************************************ *********************

**Suppl. Fig. 1. Clustal omega alignment of UBP-1 fragment I amino acids encoded by codons 1468-1562 of the *pfubp1* gene from 38 parasite lines evaluated in this study.** The variants E1528D and Y1530C are indicated.

3D7_ref KKNYLNEGNQKDDDNKNNY--------------DDKENNYDDKENNYDDKENNY--------------DDNKNNYDDNKNNY-------DDKKNNYDDKKNNY-------DDNKNNYDYNNNKNDDDDSINVSSSLKGIHKNTFDPFF

HL1211 KKNYLNEGNQKDDDNKNNYDDNKNNY-------DDKENNYDDKENNY---------------------DDNKNNYDDNKNNY-------DDKKNNYDDKKNNY-------DDNKNNYDYNNNKNDDDDSINVSSSLKGIHKNTFDPFF

HL1601 KKNYLNEGNQKDDDNKNNYDDNKNNY-------DDKENNYDDKENNY---------------------DDNKNNYDDNKNNY-------DDKKNNYDDKKNNY-------DDNKNNYDYNNNKNDDDDSINVSSSLKGIHKNTFDPFF

HL1902 KKNYLNEGNQKDDDNKNNYDDNKNNY-------DDKENNYDDKENNY--------------DDNKNNYDDNKNNYDDNKNNY-------DDKKNNYDDKKNNY-------DDNKNNYDYNNNKNDDDDSINVSSSLKGIHKNTFDPFF

HL1903 KKNYLNEGNQKDDDNKNNYDDNKNNY-------DDKENNYDDKENNY---------------------DDNKNNYDDNKNNY-------DDKKNNYDDKKNNY-------DDNKNNYDYNNNKNDDDDSINVSSSLKGIHKNTFDPFF

HL1904 KKNYLNEGNQKDDDNKNNYDDNKNNY-------DDKENNYDDKENNY--------------DDNKNNYDDNKNNYDDNKNNY-------DDKKNNYDDKKNNY-------DDNKNNYDYNNNKNDDDDSINVSSSLKGIHKNTFDPFF

HL2000 KKNYLNEGNQKDDDNKNNYDDNKNNY-------DDKENNYDDKENNY---------------------DDNKNNYDDNKNNY-------DDKKNNYDDKKNNY-------DDNKNNYDYNNNKNDDDDSINVSSSLKGIHKNTFDPFF

HL2001 KKNYLNEGNQKDDDNKNNYDDNKNNY-------DDKENNYDDKENNY---------------------DDNKNNYDDNKNNY-------DDKKNNYDDKKNNY-------DDNKNNYDYNNNKNDDDDSINVSSSLKGIHKNTFDPFF

HL2002 KKNYLNEGNQKDDDNKNNYDDNKNNY-------DDKENNYDDKENNY--------------DDNKNNYDDNKNNYDDNKNNY-------DDKKNNYDDKKNNY-------DDNKNNYDYNNNKNDDDDSINVSSSLKGIHKNTFDPFF

HL2004 KKNYLNEGNQKDDDNKNNYDDNKNNY-------DDKENNYDDKENNY---------------------DDNKNNYDDNKNNYDDKKNNYDDKKNNYDDKKNNY-------DDNKNNYDYNNNKNDDDDSINVSSSLKGIHKNTFDPFF

HL2102 KKNYLNEGNQKDDDNKNNYDDNKNNY-------DDKENNYDDKENNY---------------------DDNKNNYDDNKNNYDDKKNNYDDKKNNYDDKKNNY-------DDNKNNYDYNNNKNDDDDSINVSSSLKGIHKNTFDPFF

HL2103 KKNYLNEGNQKDDDNKNNYDDNKNNY-------DDKENNYDDKENNY---------------------DDNKNNYDDNKNNY-------DDKKNNYDDKKNNY-------DDNKNNYDYNNNKNDDDDSINVSSSLKGIHKNTFDPFF

HL2104 KKNYLNEGNQKDDDNKNNYDDNKNNY-------DDKENNYDDKENNY---------------------DDNKNNYDDNKNNY-------DDKKNNYDDKKNNY-------DDNKNNYDYNNNKNDDDDSINVSSSLKGIHKNTFDPFF

HL2105 KKNYLNEGNQKDDDNKNNYDDNKNNY-------DDKENNYDDKENNY---------------------DDNKNNYDDNKNNY-------DDKKNNYDDKKNNY-------DDNKNNYDYNNNKNDDDDSINVSSSLKGIHKNTFDPFF

HL2201 KKNYLNEGNQKDDDNKNNYDDNKNNY-------DDKENNYDDKENNY----------------------------DDNKNNYDDKKNNYDDKKNNYDDKKNNY-------DDNKNNYDYNNNKNDDDDSINVSSSLKGIHKNTFDPFF

HL2202 KKNYLNEGNQKDDDNKNNYDDNKNNY-------DDKENNYDDKENNY----------------------------DDNKNNYDDKKNNYDDKKNNYDDKKNNY-------DDNKNNYDYNNNKNDDDDSINVSSSLKGIHKNTFDPFF

HL2203 KKNYLNEGNQKDDDNKNNYDDNKNNY-------DDKENNYDDKENNY---------------------DDNKNNYDDNKNNY-------DDKKNNYDDKKNNY-------DDNKNNYDYNNNKNDDDDSINVSSSLKGIHKNTFDPFF

HL2204 KKNYLNEGNQKDDDNKNNYDDNKNNY-------DDKENNYDDKENNY---------------------DDNKNNYDDNKNNY-------DDKKNNYDDKKNNY-------DDNKNNYDYNNNKNDDDDSINVSSSLKGIHKNTFDPFF

HL2205 KKNYLNEGNQKDDDNKNNYDDNKNNY-------DDKENNYDDKENNY---------------------DDNKNNYDDNKNNYDDKKNNYDDKKNNYDDKKNNY-------DDNKNNYDYNNNKNDDDDSINVSSSLKGIHKNTFDPFF

HL2206 KKNYLNEGNQKDDDNKNNYDDNKNNY-------DDKENNYDDKENNY---------------------DDNKNNYDDNKNNYDDKKNNYDDKKNNYDDKKNNY-------DDNKNNYDYNNNKNDDDDSINVSSSLKGIHKNTFDPFF

HL2208 KKNYLNEGNQKDDDNKNNYDDNKNNYDDNKNNYDDKENNY----------------------------DDNKNNYDDNKNNY-------DDKKNNYDDKKNNY-------DDNKNNYDYNNNKNDDDDSINVSSSLKGIHKNTFDPFF

HL2209 KKNYLNEGNQKDDDNKNNYDDNKNNY-------DDKENNYDDKENNY---------------------DDNKNNYDDNKNNY-------DDKKNNYDDKKNNY-------DDNKNNYDYNNNKNDDDDSINVSSSLKGIHKNTFDPFF

HL2210 KKNYLNEGNQKDDDNKNNYDDNKNNY-------DDKENNYDDKENNY---------------------DDNKNNYDDNKNNY-------DDKKNNYDDKKNNY-------DDNKNNYDYNNNKNDDDDSINVSSSLKGIHKNTFDPFF

HL2211 KKNYLNEGNQKDDDNKNNYDDNKNNY-------DDKENNYDDKENNY--------------DDNKNNYDDNKNNYDDNKNNY-------DDKKNNYDDKKNNY-------DDNKNNYDYNNNKNDDDDSINVSSSLKGIHKNTFDPFF

HL2212 KKNYLNEGNQKDDDNKNNYDDNKNNY-------DDKENNYDDKENNY--------------DDNKNNYDDNKNNYDDNKNNY-------DDKKNNYDDKKNNY-------DDNKNNYDYNNNKNDDDDSINVSSSLKGIHKNTFDPFF

HL2213 KKNYLNEGNQKDDDNKNNYDDNKNNY-------DDKENNYDDKENNY---------------------DDNKNNYDDNKNNY-------DDKKNNYDDKKNNY-------DDNKNNYDYNNNKNDDDDSINVSSSLKGIHKNTFDPFF

HL2214 KKNYLNEGNQKDDDNKNNYDDNKNNY-------DDKENNYDDKKNNY-------DDNKNNYDDNKNNYDDNKNNYDDNKNNY-------DDKKNNYDDKKNNY-------DDNKNNYDYNNNKNDDDDSINVSSSLKGIHKNTFDPFF

HL2301 KKNYLNEGNQKDDDNKNNYDDNKNNY-------DDKENNYDDN**K**NNYDDKENNY--------------DDNKNNYDDNKNNY-------DDKKNNYDDKKNNY-------DDNKNNYDYNNNKNDDDDSINVSSSLKGIHKNTFDPFF

HL2302 KKNYLNEGNQKDDDNKNNYDDNKNNYDDNKNNYDDKENNYDDKENNY---------------------DDNKNNYDDNKNNY-------DDKKNNYDDKKNNY-------DDNKNNYDYNNNKNDDDDSINVSSSLKGIHKNTFDPFF

HL2303 KKNYLNERNQKDDDNKNNYDDNKNNYDDNKNNYDDKENNYDDKENNY---------------------DDNKNNYDDNKNNY-------DDKKNNYDDKKNNY-------DDNKNNYDYNNNKNDDDDSINVSSSLKGIHKNTFDPFF

HL2304 KKNYLNEGNQKDDDNKNNYDDNKNNY-------DDKENNYDDKENNY---------------------DDNKNNYDDNKNNYDDKKNNYDDKKNNYDDKKNNYDDNKNNYDDNKNNYDYNNNKNDDDDSINVSSSLKGIHKNTFDPFF

HL2305 KKNYLNEGNQKDDDNKNNYDDNKNNY-------DDKENNYDDKKNNY--------------DDNKNNYDDNKNNYDDNKNNY--------------DDKKNNYDDNKNNYDDNKNNYDYNNNKNDDDDSINVSSSLKGIHKNTFDPFF

HL2306 KKNYLNEGNQKDDDNKNNYDDNKNNYDDNKNNYDDKENNYDDKENNY---------------------DDNKNNYDDNKNNY-------DDKKNNYDDKKNNY-------DDNKNNYDYNNNKNDDDDSINVSSSLKGIHKNTFDPFF

HL2307 KKNYLNEGNQKDDDNKNNYDDNKNNY-------DDKENNYDDKENNY---------------------DDNKNNYDDNKNNY-------DDKKNNYDDKKNNY-------DDNKNNYDYNNNKNDDDDSINVSSSLKGIHKNTFDPFF

HL2308 KKNYLNEGNQKDDDNKNNYDDNKNNY-------DDKENNYDDKENNY---------------------DDNKNNYDDNKNNY-------DDKKNNYDDKKNNY-------DDNKNNYDYNNNKNDDDDSINVSSSLKGIHKNTFDPFF

HL2309 KKNYLNEGNQKDDDNKNNYDDNKNNY-------DDKENNYDDKENNY--------------DDNKNNYDDNKNNYDDNKNNY--------------DDKKNNY-------DDNKNNYDYNNNKNDDDDSINVSSSLKGIHKNTFDPFF

******* ****************** *********:.*** ************** ************** **************************************

3D7 EKHSNNSLMDSGDDYLCDMNNLSNNKKDIYILWTYFESSKCVGYNECKTLLSLCLKNENETCINNISASKLRSLVISIWSNIPSSKPKRSFIKLIFNWINNKKDDLHKKKNLFYLLKSEKKNNKNLSKICFNYFLNYLIKYKDNCSNDI

HL1211 EKHSNNSLMDSGDDYLCDMNNLSNNKKDIYILWTYFESSKCVGYNECKTLLSLCLKNENETCINNISASKLRSLVISIWSNIPSSKPKRSFIKLIFNWINNKKDDLHKKKNLFYLLKSEKKNNKNLSKICFNYFLNYLIKYKDNCSNDI

HL1601 EKHSNNSLMDSGDDYLCDMNNLSNNKKDIYILWTYFESSKCVGYNECKTLLSLCLKNENETCINNISASKLRSLVISIWSNIPSSKPKRSFIKLIFNWINNKKDDLHKKKNLFYLLKSEKKNNKNLSKICFNYFLNYLIKYKDNCSNDI

HL1902 EKHSNNSLMDSGDDYLCDMNNLSNNKKDIYILWTYFESSKCVGYNECKTLLSLCLKNENETCINNISASKLRSLVISIWSNIPSSKPKRSFIKLIFNWINNKKDDLHKKKNLFYLLKSEKKNNKNLSKICFNYFLNYLIKYKDNCSNDI

HL1903 EKHSNNSLMDSGDDYLCDMNNLSNNKKDIYILWTYFESSKCVGYNECKTLLSLCLKNENETCINNISASKLRSLVISIWSNIPSSKPKRSFIKLIFNWINNKKDDLHKKKNLFYLLKSEKKNNKNLSKICFNYFLNYLIKYKDNCSNDI

HL1904 EKHSNNSLMDSGDDYLCDMNNLSNNKKDIYILWTYFESSKCVGYNECKTLLSLCLKNENETCINNISASKLRSLVISIWSNIPSSKPKRSFIKLIFNWINNKKDDLHKKKNLFYLLKSEKKNNKNLSKICFNYFLNYLIKYKDNCSNDI

HL2000 EKHSNNSLMDSGDDYLCDMNNLSNNKKDIYILWTYFESSKCVGYNECKTLLSLCLKNENETCINNISASKLRSLVISIWSNIPSSKPKRSFIKLIFNWINNKKDDLHKKKNLFYLLKSEKKNNKNLSKICFNYFLNYLIKYKDNCSNDI

HL2001 EKHSNNSLMDSGDDYLCDMNNLSNNKKDIYILWTYFESSKCVGYNECKTLLSLCLKNENETCINNISASKLRSLVISIWSNIPSSKPKRSFIKLIFNWINNKKDDLHKKKNLFYLLKSEKKNNKNLSKICFNYFLNYLIKYKDNCSNDI

HL2002 EKHSNNSLMDSGDDYLCDMNNLSNNKKDIYILWTYFESSKCVGYNECKTLLSLCLKNENETCINNISASKLRSLVISIWSNIPSSKPKRSFIKLIFNWINNKKDDLHKKKNLFYLLKSEKKNNKNLSKICFNYFLNYLIKYKDNCSNDI

HL2004 EKHSNNSLMDSGDDYLCDMNNLSNNKKDIYILWTYFESSKCVGYNECKTLLSLCLKNENETCINNISASKLRSLVISIWSNIPSSKPKRSFIKLIFNWINNKKDDLHKKKNLFYLLKSEKKNNKNLSKICFNYFLNYLIKYKDNCSNDI

HL2102 EKHSNNSLMDSGDDYLCDMNNLSNNKKDIYILWTYFESSKCVGYNECKTLLSLCLKNENETCINNISASKLRSLVISIWSNIPSSKPKRSFIKLIFNWINNKKDDLHKKKNLFYLLKSEKKNNKNLSKICFNYFLNYLIKYKDNCSNDI

HL2103 EKHSNNSLMDSGDDYLCDMNNLSNNKKDIYILWTYFESSKCVGYNECKTLLSLCLKNENETCINNISASKLRSLVISIWSNIPSSKPKRSFIKLIFNWINNKKDDLHKKKNLFYLLKSEKKNNKNLSKICFNYFLNYLIKYKDNCSNDI

HL2104 EKHSNNSLMDSGDDYLCDMNNLSNNKKDIYILWTYFESSKCVGYNECKTLLSLCLKNENETCINNISASKLRSLVISIWSNIPSSKPKRSFIKLIFNWINNKKDDLHKKKNLFYLLKSEKKNNKNLSKICFNYFLNYLIKYKDNCSNDI

HL2105 EKHSNNSLMDSGDDYLCDMNNLSNNKKDIYILWTYFESSKCVGYNECKTLLSLCLKNENETCINNISASKLRSLVISIWSNIPSSKPKRSFIKLIFNWINNKKDDLHKKKNLFYLLKSEKKNNKNLSKICFNYFLNYLIKYKDNCSNDI

HL2201 EKHSNNSLMDSGDDYLCDMNNLSNNKKDIYILWTYFESSKCVGYNECKTLLSLCLKNENETCINNISASKLRSLVISIWSNIPSSKPKRSFIKLIFNWINNKKDDLHKKKNLFYLLKSEKKNNKNLSKICFNYFLNYLIKYKDNCSNDI

HL2202 EKHSNNSLMDSGDDYLCDMNNLSNNKKDIYILWTYFESSKCVGYNECKTLLSLCLKNENETCINNISASKLRSLVISIWSNIPSSKPKRSFIKLIFNWINNKKDDLHKKKNLFYLLKSEKKNNKNLSKICFNYFLNYLIKYKDNCSNDI

HL2203 EKHSNNSLMDSGDDYLCDMNNLSNNKKDIYILWTYFESSKCVGYNECKTLLSLCLKNENETCINNISASKLRSLVISIWSNIPSSKPKRSFIKLIFNWINNKKDDLHKKKNLFYLLKSEKKNNKNLSKICFNYFLNYLIKYKDNCSNDI

HL2204 EKHSNNSLMDSGDDYLCDMNNLSNNKKDIYILWTYFESSKCVGYNECKTLLSLCLKNENETCINNISASKLRSLVISIWSNIPSSKPKRSFIKLIFNWINNKKDDLHKKKNLFYLLKSEKKNNKNLSKICFNYFLNYLIKYKDNCSNDI

HL2205 EKHSNNSLMDSGDDYLCDMNNLSNNKKDIYILWTYFESSKCVGYNECKTLLSLCLKNENETCINNISASKLRSLVISIWSNIPSSKPKRSFIKLIFNWINNKKDDLHKKKNLFYLLKSEKKNNKNLSKICFNYFLNYLIKYKDNCSNDI

HL2206 EKHSNNSLMDSGDDYLCDMNNLSNNKKDIYILWTYFESSKCVGYNECKTLLSLCLKNENETCINNISASKLRSLVISIWSNIPSSKPKRSFIKLIFNWINNKKDDLHKKKNLFYLLKSEKKNNKNLSKICFNYFLNYLIKYKDNCSNDI

HL2208 EKHSNNSLMDSGDDYLCDMNNLSNNKKDIYILWTYFESSKCVGYNECKTLLSLCLKNENETCINNISASKLRSLVISIWSNIPSSKPKRSFIKLIFNWINNKKDDLHKKKNLFYLLKSEKKNNKNLSKICFNYFLNYLIKYKDNCSNDI

HL2209 EKHSNNSLMDSGDDYLCDMNNLSNNKKDIYILWTYFESSKCVGYNECKTLLSLCLKNENETCINNISASKLRSLVISIWSNIPSSKPKRSFIKLIFNWINNKKDDLHKKKNLFYLLKSEKKNNKNLSKICFNYFLNYLIKYKDNCSNDI

HL2210 EKHSNNSLMDSGDDYLCDMNNLSNNKKDIYILWTYFESSKCVGYNECKTLLSLCLKNENETCINNISASKLRSLVISIWSNIPSSKPKRSFIKLIFNWINNKKDDLHKKKNLFYLLKSEKKNNKNLSKICFNYFLNYLIKYKDNCSNDI

HL2211 EKHSNNSLMDSGDDYLCDMNNLSNNKKDIYILWTYFESSKCVGYNECKTLLSLCLKNENETCINNISASKLRSLVISIWSNIPSSKPKRSFIKLIFNWINNKKDDLHKKKNLFYLLKSEKKNNKNLSKICFNYFLNYLIKYKDNCSNDI

HL2212 EKHSNNSLMDSGDDYLCDMNNLSNNKKDIYILWTYFESSKCVGYNECKTLLSLCLKNENETCINNISASKLRSLVISIWSNIPSSKPKRSFIKLIFNWINNKKDDLHKKKNLFYLLKSEKKNNKNLSKICFNYFLNYLIKYKDNCSNDI

HL2213 EKHSNNSLMDSGDDYLCDMNNLSNNKKDIYILWTYFESSKCVGYNECKTLLSLCLKNENETCINNISASKLRSLVISIWSNIPSSKPKRSFIKLIFNWINNKKDDLHKKKNLFYLLKSEKKNNKNLSKICFNYFLNYLIKYKDNCSNDI

HL2214 EKHSNNSLMDSGDDYLCDMNNLSNNKKDIYILWTYFESSKCVGYNECKTLLSLCLKNENETCINNISASKLRSLVISIWSNIPSSKPKRSFIKLIFNWINNKKDDLHKKKNLFYLLKSEKKNNKNLSKICFNYFLNYLIKYKDNCSNDI

HL2301 EKHSNNSLMDSGDDYLCDMNNLSNNKKDIYILWTYFESSKCVGYNECKTLLSLCLKNENETCINNISASKLRSLVISIWSNIPSSKPKRSFIKLIFNWINNKKDDLHKKKNLFYLLKSEKKNNKNLSKICFNYFLNYLIKYKDNCSNDI

HL2302 EKHSNNSLMDSGDDYLCDMNNLSNNKKDIYILWTYFESSKCVGYNECKTLLSLCLKNENETCINNISASKLRSLVISIWSNIPSSKPKRSFIKLIFNWINNKKDDLHKKKNLFYLLKSEKKNNKNLSKICFNYFLNYLIKYKDNCSNDI

HL2303 EKHSNNSLMDSGDDYLCDMNNLSNNKKDIYILWTYFESSKCVGYNECKTLLSLCLKNENETCINNISASKLRSLVISIWSNIPSSKPKRSFIKLIFNWINNKKDDLHKKKNLFYLLKSEKKNNKNLSKICFNYFLNYLIKYKDNCSNDI

HL2304 EKHSNNSLMDSGDDYLCDMNNLSNNKKDIYILWTYFESSKCVGYNECKTLLSLCLKNENETCINNISASKLRSLVISIWSNIPSSKPKRSFIKLIFNWINNKKDDLHKKKNLFYLLKSEKKNNKNLSKICFNYFLNYLIKYKDNCSNDI

HL2305 EKHSNNSLMDSGDDYLCDMNNLSNNKKDIYILWTYFESSKCVGYNECKTLLSLCLKNENETCINNISASKLRSLVISIWSNIPSSKPKRSFIKLIFNWINNKKDDLHKKKNLFYLLKSEKKNNKNLSKICFNYFLNYLIKYKDNCSNDI

HL2306 EKHSNNSLMDSGDDYLCDMNNLSNNKKDIYILWTYFESSKCVGYNECKTLLSLCLKNENETCINNISASKLRSLVISIWSNIPSSKPKRSFIKLIFNWINNKKDDLHKKKNLFYLLKSEKKNNKNLSKICFNYFLNYLIKYKDNCSNDI

HL2307 EKHSNNSLMDSGDDYLCDMNNLSNNKKDIYILWTYFESSKCVGYNECKTLLSLCLKNENETCINNISASKLRSLVISIWSNIPSSKPKRSFIKLIFNWINNKKDDLHKKKNLFYLLKSEKKNNKNLSKICFNYFLNYLIKYKDNCSNDI

HL2308 EKHSNNSLMDSGDDYLCDMNNLSNNKKDIYILWTYFESSKCVGYNECKTLLSLCLKNENETCINNISASKLRSLVISIWSNIPSSKPKRSFIKLIFNWINNKKDDLHKKKNLFYLLKSEKKNNKNLSKICFNYFLNYLIKYKDNCSNDI

HL2309 EKHSNNSLMDSGDDYLCDMNNLSNNKKDIYILWTYFESSKCVGYNECKTLLSLCLKNENETCINNISASKLRSLVISIWSNIPSSKPKRSFIKLIFNWINNKKDDLHKKKNLFYLLKSEKKNNKNLSKICFNYFLNYLIKYKDNCSNDI

*****************************************************************************************************************************************************

3D7 IYILYLIDENELKIYSKNFIQNHKINFNQFISIWNIMCILFWDTDEINNFTFLQKNKYYYYDFMLIFLKTFYDYINVNRDMREIMKMKLK**R**TFLTGYHHDVEEPSQEHMSLYQEKNNIHNQDNRLSFTYMKKMSLSNSSINNKQDKHEDQN

HL1211 IYILYLIDENELKIYSKNFIQNHKINFNQFISIWNIMCILFWDTDEINNFTFLQKNKYYYYDFMLIFLKTFYDYINVNRDMREIMKMKLKKTFLTGYHHDVEEPSQEHMSLYQEKNNIHNQDNRLSFTYMKKM**P**LSNSSINNKQDKHEDQN

HL1601 IYILYLIDENELKIYSKNFIQNHKINFNQFISIWNIMCILFWDTDEINNFTFLQKNKYYYYDFMLIFLKTFYDYINVNRDMREIMKMKLKKTFLTGYHHDVEEPSQEHMSLYQEKNNIHNQDNRLSFTYMKKMSLSNSSINNKQDKHEDQN

HL1902 IYILYLIDENELKIYSKNFIQNHKINFNQFISIWNIMCILFWDTDEINNFTFLQKNKYYYYDFMLIFLKTFYDYINVNRDMREIMKMKLKKTFLTGYHHDVEEPSQEHMSLYQEKNNIHNQDNRLSFTYMKKMSLSNSSINNKQDKHEDQN

HL1903 IYILYLIDENELKIYSKNFIQNHKINFNQFISIWNIMCILFWDTDEINNFTFLQKNKYYYYDFMLIFLKTFYDYINVNRDMREIMKMKLKKTFLTGYHHDVEEPSQEHMSLYQEKNNIHNQDNRLSFTYMKKMSLSNSSINNKQDKHEDQN

HL1904 IYILYLIDENELKIYSKNFIQNHKINFNQFISIWNIMCILFWDTDEINNFTFLQKNKYYYYDFMLIFLKTFYDYINVNRDMREIMKMKLKKTFLTGYHHDVEEPSQEHMSLYQEKNNIHNQDNRLSFTYMKKMSLSNSSINNKQDKHEDQN

HL2000 IYILYLIDENELKIYSKNFIQNHKINFNQFISIWNIMCILFWDTDEINNFTFLQKNKYYYYDFMLIFLKTFYDYINVNRDMREIMKMKLK**R**TFLTGYHHDVEEPSQEHMSLYQEKNNIHNQDNRLSFTYMKKMSLSNSSINNKQDKHEDQN

HL2001 IYILYLIDENELKIYSKNFIQNHKINFNQFISIWNIMCILFWDTDEINNFTFLQKNKYYYYDFMLIFLKTFYDYINVNRDMREIMKMKLK**R**TFLTGYHHDVEEPSQEHMSLYQEKNNIHNQDNRLSFTYMKKMSLSNSSINNKQDKHEDQN

HL2002 IYILYLIDENELKIYSKNFIQNHKINFNQFISIWNIMCILFWDTDEINNFTFLQKNKYYYYDFMLIFLKTFYDYINVNRDMREIMKMKLKKTFLTGYHHDVEEPSQEHMSLYQEKNNIHNQDNRLSFTYMKKMSLSNSSINNKQDKHEDQN

HL2004 IYILYLIDENELKIYSKNFIQNHKINFNQFISIWNIMCILFWDTDEINNFTFLQKNKYYYYDFMLIFLKTFYDYINVNRDMREIMKMKLKKTFLTGYHHDVEEPSQEHMSLYQEKNNIHNQDNRLSFTYMKKMSLSNSSINNKQDKHEDQN

HL2102 IYILYLIDENELKIYSKNFIQNHKINFNQFISIWNIMCILFWDTDEINNFTFLQKNKYYYYDFMLIFLKTFYDYINVNRDMREIMKMKLKKTFLTGYHHDVEEPSQEHMSLYQEKNNIHNQDNRLSFTYMKKMSLSNSSINNKQDKHEDQN

HL2103 IYILYLIDENELKIYSKNFIQNHKINFNQFISIWNIMCILFWDTDEINNFTFLQKNKYYYYDFMLIFLKTFYDYINVNRDMREIMKMKLKKTFLTGYHHDVEEPSQEHMSLYQEKNNIHNQDNRLSFTYMKKMSLSNSSINNKQDKHEDQN

HL2104 IYILYLIDENELKIYSKNFIQNHKINFNQFISIWNIMCILFWDTDEINNFTFLQKNKYYYYDFMLIFLKTFYDYINVNRDMREIMKMKLK**R**TFLTGYHHDVEEPSQEHMSLYQEKNNIHNQDNRLSFTYMKKMSLSNSSINNKQDKHEDQN

HL2105 IYILYLIDENELKIYSKNFIQNHKINFNQFISIWNIMCILFWDTDEINNFTFLQKNKYYYYDFMLIFLKTFYDYINVNRDMREIMKMKLKKTFLTGYHHDVEEPSQEHMSLYQEKNNIHNQDNRLSFTYMKKMSLSNSSINNKQDKHEDQN

HL2201 IYILYLIDENELKIYSKNFIQNHKINFNQFISIWNIMCILFWDTDEINNFTFLQKNKYYYYDFMLIFLKTFYDYINVNRDMREIMKMKLKKTFLTGYHHDVEEPSQEHMSLYQEKNNIHNQDNRLSFTYMKKMSLSNSSINNKQDKHEDQN

HL2202 IYILYLIDENELKIYSKNFIQNHKINFNQFISIWNIMCILFWDTDEINNFTFLQKNKYYYYDFMLIFLKTFYDYINVNRDMREIMKMKLKKTFLTGYHHDVEEPSQEHMSLYQEKNNIHNQDNRLSFTYMKKMSLSNSSINNKQDKHEDQN

HL2203 IYILYLIDENELKIYSKNFIQNHKINFNQFISIWNIMCILFWDTDEINNFTFLQKNKYYYYDFMLIFLKTFYDYINVNRDMREIMKMKLKKTFLTGYHHDVEEPSQEHMSLYQEKNNIHNQDNRLSFTYMKKMSLSNSSINNKQDKHEDQN

HL2204 IYILYLIDENELKIYSKNFIQNHKINFNQFISIWNIMCILFWDTDEINNFTFLQKNKYYYYDFMLIFLKTFYDYINVNRDMREIMKMKLKKTFLTGYHHDVEEPSQEHMSLYQEKNNIHNQDNRLSFTYMKKMSLSNSSINNKQDKHEDQN

HL2205 IYILYLIDENELKIYSKNFIQNHKINFNQFISIWNIMCILFWDTDEINNFTFLQKNKYYYYDFMLIFLKTFYDYINVN**K**DMREIMKMKLKKTFLTGYHHDVEEPSQEHMSLYQEKNNIHNQDNRLSFTYMKKMSLSNSSINNKQDKHEDQN

HL2206 IYILYLIDENELKIYSKNFIQNHKINFNQFISIWNIMCILFWDTDEINNFTFLQKNKYYYYDFMLIFLKTFYDYINVNRDMREIMKMKLKKTFLTGYHHDVEEPSQEHMSLYQEKNNIHNQDNRLSFTYMKKMSLSNSSINNKQDKHEDQN

HL2208 IYILYLIDENELKIYSKNFIQNHKINFNQFISIWNIMCILFWDTDEINNFTFLQKNKYYYYDFMLIFLKTFYDYINVNRDMREIMKMKLKKTFLTGYHHDVEEPSQEHMSLYQEKNNIHNQDNRLSFTYMKKMSLSNSSINNKQDKHEDQN

HL2209 IYILYLIDENELKIYSKNFIQNHKINFNQFISIWNIMCILFWDTDEINNFTFLQKNKYYYYDFMLIFLKTFYDYINVNRDMREIMKMKLKKTFLTGYHHDVEEPSQEHMSLYQEKNNIHNQDNRLSFTYMKKMSLSNSSINNKQDKHEDQN

HL2210 IYILYLIDENELKIYSKNFIQNHKINFNQFISIWNIMCILFWDTDEINNFTFLQKNKYYYYDFMLIFLKTFYDYINVNRDMREIMKMKLKKTFLTGYHHDVEEPSQEHMSLYQEKNNIHNQDNRLSFTYMKKMSLSNSSINNKQDKHEDQN

HL2211 IYILYLIDENELKIYSKNFIQNHKINFNQFISIWNIMCILFWDTDKINNFTFIQKNKYYYYDFMLIFLKTFYDYINVNRDMREIMKMKLKKTFLTGYHHDVEEPSQEHMSLYQEKNNIHNQDNRLSFTYMKKMSLSNSSINNKQDKHEDQN

HL2212 IYILYLIDENELKIYSKNFIQNHKINFNQFISIWNIMCILFWDTDEINNFTFLQKNKYYYYDFMLIFLKTFYDYINVNRDMREIMKMKLKKTFLTGYHHDVEEPSQEHMSLYQEKNNIHNQDNRLSFTYMKKMSLSNSSINNKQDKHEDQN

HL2213 IYILYLIDENELKIYSKNFIQNHKINFNQFISIWNIMCILFWDTDEINNFTFLQKNKYYYYDFMLIFLKTFYDYINVNRDMREIMKMKLKKTFLTGYHHDVEEPSQEHMSLYQEKNNIHNQDNRLSFTYMKKMSLSNSSINNKQDKHEDQN

HL2214 IYILYLIDENELKIYSKNFIQNHKINFNQFISIWNIMCILFWDTDEINNFTFLQKNKYYYYDFMLIFLKTFYDYINVNRDMREIMKMKLKKTFLTGYHHDVEEPSQEHMSLYQEKNNIHNQDNRLSFTYMKKMSLSNSSINNKQDKHEDQN

HL2301 IYILYLIDENELKIYSKNFIQNHKINFNQFISIWNIMCILFWDTDEINNFTFLQKNKYYYYDFMLIFLKTFYDYINVNRDMREIMKMKLKKTFLTGYHHDVEEPSQEHMSLYQEKNNIHNQDNRLSFTYMKKMSLSNSSINNKQDKHEDQN

HL2302 IYILYLIDENELKIYSKNFIQNHKINFNQFISIWNIMCILFWDTDEINNFTFLQKNKYYYYDFMLIFLKTFYDYINVNRDMREIMKMKLKKTFLTGYHHDVEEPSQEHMSLYQEKNNIHNQDNRLSFTYMKKMSLSNSSINNKQDKHEDQN

HL2303 IYILYLIDENELKIYSKNFIQNHKINFNQFISIWNIMCILFWDTDEINNFTFLQKNKYYYYDFMLIFLKTFYDYINVNRDMREIMKMKLK**R**TFLTGYHHDVEEPSQEHMSLYQEKNNIHNQDNRLSFTYMKKMSLSNSSINNKQDKHEDQN

HL2304 IYILYLIDENELKIYSKNFIQNHKINFNQFISIWNIMCILFWDTDEINNFTFLQKNKYYYYDFMLIFLKTFYDYINVNRDMREIMKMKLKKTFLTGYHHDVEEPSQEHMSLYQEKNNIHNQDNRLSFTYMKKMSLSNSSINNKQDKHEDQN

HL2305 IYILYLIDENELKIYSKNFIQNHKINFNQFISIWNIMCILFWDTDEINNFTFLQKNKYYYYDFMLIFLKTFYDYINVNRDMREIMKMKLKKTFLTGYHHDVEEPSQEHMSLYQEKNNIHNQDNRLSFTYMKKMSLSNSSINNKQDKHEDQN

HL2306 IYILYLIDENELKIYSKNFIQNHKINFNQFISIWNIMCILFWDTDEINNFTFLQKNKYYYYDFMLIFLKTFYDYINVNRDMREIMKMKLKKTFLTGYHHDVEEPSQEHMSLYQEKNNIHNQDNRLSFTYMKKMSLSNSSINNKQDKHEDQN

HL2307 IYILYLIDENELKIYSKNFIQNHKINFNQFISIWNIMCILFWDTDEINNFTFLQKNKYYYYDFMLIFLKTFYDYINVNRDMREIMKMKLK**R**TFLTGYHHDVEEPSQEHMSLYQEKNNIHNQDNRLSFTYMKKMSLSNSSINNKQDKHEDQN

HL2308 IYILYLIDENELKIYSKNFIQNHKINFNQFISIWNIMCILFWDTDEINNFTFLQKNKYYYYDFMLIFLKTFYDYINVNRDMREIMKMKLK**R**TFLTGYHHDVEEPSQEHMSLYQEKNNIHNQDNRLSFTYMKKMSLSNSSINNKQDKHEDQN

HL2309 IYILYLIDENELKIYSKNFIQNHKINFNQFISIWNIMCILFWDTDEINNFTFLQKNKYYYYDFMLIFLKTFYDYINVNRDMREIMKMKLKKTFLTGYHHDVEEPSQEHMSLYQEKNNIHNQDNRLSFTYMKKMSLSNSSINNKQDKHEDQN

*********************************************:******:*************************:***********:****************************************** *****************

**Suppl. Fig. 2. Clustal omega alignment of UBP-1 fragment II amino acids encoded by codons 1893 – 2339 of the *pfubp1* gene from 36 parasite lines evaluated in this study.**

3D7 GVSNQNMNERTYAENLHNMNNIHNNKFCPSSYRHTQNILNMNSTHNNSSVNNNFNKMNHSISEKMGKNKNDNIFSFLKSTKNNMSFDQNGRLVNSNINYMKNKNLLLCKEEQEKHTSFQSLNCNRTKNNSIQERVVYGKEINNNHNLKDINVFKYKKHEHKHGEFFNLNNMK

HL1211 GVSNQNMNERTYAENLHNMNNIHNNKFCPSSYRHTQNILNMNSTHNNSSVNNNFNKMNHSISEKMGKNKNDNIFSFLKSTKNNMSFDQNGRLVNSNINYMKNKNLLLCKEEQEKHTSFQSLNCNRTKNNSIQERVVYGKEINNNHNLKDINVFKYKKHEHKHGEFFNLNNMK

HL1601 GVSNQNMNERTYAENLHNMNNIHNNKFCPSSYRHTQNILNMNSTHNNSSVNNNFNKMNHSISEKMGKNKNDNIFSFLKSTKNNMSFDQNGRLVNSNINYMKNKNLLLCKEEQEKHTSFQSLNCNRTKNNSIQERVVYGKEINNNHNLKDINVFKYKKHEHKHGEFFNLNNMK

HL1901 GVSNQNMNERTYAENLHNMNNIHNNKFCPSSYRHTQNILNMNSTHNNSSVNNNFNKMNHSISEKMGKNKNDNIFSFLKSTKNNMSFDQNGRLVNSNINYMKNKNLLLCKEEQEKHTSFQSLNCNRTKNNSIQERVVYGKEINNNHNLKDINVFKYKKHEHKHGEFFNLNNMK

HL1902 GVSNQNMNERTYAENLHNMNNIHNNKFCPSSYRHTQNILNMNSTHNNSSVNNNFNKMNHSISEKMGKNKNDNIFSFLKSTKNNMSFDQNGRLVNSNINYMKNKNLLLCKEEQEKHTSFQSLNCNRTKNNSIQERVVYGKEINNNHNLKDINVFKYKKHEHKHGEFFNLNNMK

HL1903 GVSNQNMNERTYAENLHNMNNIHNNKFCPSSYRHTQNILNMNSTHNNSSVNNNFNKMNHSISEKMGKNKNDNIFSFLKSTKNNMSFDQNGRLVNSNINYMKNKNLLLCKEEQEKHTSFQSLNCNRTKNNSIQERVVYGKEINNNHNLKDINVFKYKKHEHKHGEFFNLNNMK

HL1904 GVSNQNMNERTYAENLHNMNNIHNNKFCPSSYRHTQNILNMNSTHNNSSVNNNFNKMNHSISEKMGKNKNDNIFSFLKSTKNNMSFDQNGRLVNSNINYMKNKNLLLCKEEQEKHTSFQSLNCNRTKNNSIQERVVYGKEINNNHNLKDINVFKYKKHEHKHGEFFNLNNMK

HL2000 GVSNQNMNERTYAENLHNMNNIHNNKFCPSSYRHTQNILNMNSTHNNSSVNNNFNKMNHSISEKMGKNKNDNIFSFLKSTKNNMSFDQNGRLVNSNINYMKNKNLLLCKEEQEKHTSFQSLNCNRTKNNSIQERVVYGKEINNNHNLKDINVFKYKKHEHKHGEFFNLNNMK

HL2001 GVSNQNMNERTYAENLHNMNNIHNNKFCPSSYRHTQNILNMNSTHNNSSVNNNFNKMNHSISEKMGKNKNDNIFSFLKSTKNNMSFDQNGRLVNSNINYMKNKNLLLCKEEQEKHTSFQSLNCNRTKNNSIQERVVYGKEINNNHNLKDINVFKYKKHEHKHGEFFNLNNMK

HL2002 GVSNQNMNERTYAENLHNMNNIHNNKFCPSSYRHTQNILNMNSTHNNSSVNNNFNKMNHSISEKMGKNKNDNIFSFLKSTKNNMSFDQNGRLVNSNINYMKNKNLLLCKEEQEKHTSFQSLNCNRTKNNSIQERVVYGKEINNNHNLKDINVFKYKKHEHKHGEFFNLNNMK

HL2004 GVSNQNMNERTYAENLHNMNNIHNNKFCPSSYRHTQNILNMNSTHNNSSVNNNFNKMNHSISEKMGKNKNDNIFSFLKSTKNNMSFDQNGRLVNSNINYMKNKNLLLCKEEQEKHTSFQSLNCNRTKNNSIQERVVYGKEINNNHNLKDINVFKYKKHEHKHGEFFNLNNMK

HL2102 GVSNQNMNERTYAENLHNMNNIHNNKFCPSSYRHTQNILNMNSTHNNSSVNNNFNKMNHSISEKMGKNKNDNIFSFLKSTKNNMSFDQNGRLVNSNINYMKNKNLLLCKEEQEKHTSFQSLNCNRTKNNSIQERVVYGKEINNNHNLKDINVFKYKKHEHKHGEFFNLNNMK

HL2103 GVSNQNMNERTYAENLHNMNNIHNNKFCPSSYRHTQNILNMNSTHNNSSVNNNFNKMNHSISEKMGKNKNDNIFSFLKSTKNNMSFDQNGRLVNSNINYMKNKNLLLCKEEQEKHTSFQSLNCNRTKNNSIQERVVYGKEINNNHNLKDINVFKYKKHEHKHGEFFNLNNMK

HL2104 GVSNQNMNERTYAENLHNMNNIHNNKFCPSSYRHTQNILNMNSTHNNSSVNNNFNKMNHSISEKMGKNKNDNIFSFLKSTKNNMSFDQNGRLVNSNINYMKNKNLLLCKEEQEKHTSFQSLNCNRTKNNSIQERVVYGKEINNNHNLKDINVFKYKKHEHKHGEFFNLNNMK

HL2105 GVSNQNMNERTYAENLHNMNNIHNNKFCPSSYRHTQNILNMNSTHNNSSVNNNFNKMNHSISEKMGKNKNDNIFSFLKSTKNNMSFDQNGRLVNSNINYMKNKNLLLCKEEQEKHTSFQSLNCNRTKNNSIQERVVYGKEINNNHNLKDINVFKYKKHEHKHGEFFNLNNMK

HL2201 GVSNQNMNERTYAENLHNMNNIHNNKFCPSSYRHTQNILNMNSTHNNSSVNNNFNKMNHSISEKMGKNKNDNIFSFLKSTKNNMSFDQNGRLVNSNINYMKNKNLLLCKEEQEKHTCFQSLNCNRTKNNSIQERVVYGKEINNN**R**NLKDINVFKYKKHEHKHGEFFNLNNMK

HL2202 GVSNQNMNERTYAENLHNMNNIHNNKFCPSSYRHTQNILNMNSTHNNSSVNNNFNKMNHSISEKMGKNKNDNIFSFLKSTKNNMSFDQNGRLVNSNINYMKNKNLLLCKEEQEKHTCFQSLNCNRTKNNSIQERVVYGKEINNN**R**NLKDINVFKYKKHEHKHGEFFNLNNMK

HL2203 GVSNQNMNERTYAENLHNMNNIHNNKFCPSSYRHTQNILNMNSTHNNSSVNNNFNKMNHSISEKMGKNKNDNIFSFLKSTKNNMSFDQNGRLVNSNINYMKNKNLLLCKEEQEKHTSFQSLNCNRTKNNSIQERVVYGKEINNNHNLKDINVFKYKKHEHKHGEFFNLNNMK

HL2204 GVSNQNMNERTYAENLHNMNNIHNNKFCPSSYRHTQNILNMNSTHNNSSVNNNFNKMNHSISEKMGKNKNDNIFSFLKSTKNNMSFDQNGRLVNSNINYMKNKNLLLCKEEQEKHTSFQSLNCNRTKNNSIQERVVYGKEINNNHNLKDINVFKYKKHEHKHGEFFNLNNMK

HL2205 GVSNQNMNERTYAENLHNMNNIHNNKFCPSSYRHTQNILNMNSTHNNSSVNNNFNKMNHSISEKMGKNKNDNIFSFLKSTKNNMSFDQNGRLVNSNINYMKNKNLLLCKEEQEKHTSFQSLNCNRTKNNSIQERVVYGKEINNNHNLKDINVFKYKKHEHKHGEFFNLNNMK

HL2206 GVSNQNMNERTYAENLHNMNNIHNNKFCPSSYRHTQNILNMNSTHNNSSVNNNFNKMNHSISEKMGKNKNDNIFSFLKSTKNNMSFDQNGRLVNSNINYMKNKNLLLCKEEQEKHTSFQSLNCNRTKNNSIQERVVYGKEINNNHNLKDINVFKYKKHEHKHGEFFNLNNMK

HL2208 GVSNQNMNERTYAENLHNMNNIHNNKFCPSSYRHTQNILNMNSTHNNSSVNNNFNKMNHSISEKMGKNKNDNIFSFLKSTKNNMSFDQNGRLVNSNINYMKNKNLLLCKEEQEKHTSFQSLNCNRTKNNSIQERVVYGKEINNNHNLKDINVFKYKKHEHKHGEFFNLNNMK

HL2209 GVSNQNMNERTYAENLHNMNNIHNNKFCPSSYRHTQNILNMNSTHNNSSVNNNFNKMNHSISEKMGKNKNDNIFSFLKSTKNNMSFDQNGRLVNSNINYMKNKNLLLCKEEQEKHTSFQSLNCNRTKNNSIQERVVYGKEINNNHNLKDINVFKYKKHEHKHGEFFNLNNMK

HL2210 GVSNQNMNERTYAENLHNMNNIHNNKFCPSSYRHTQNILNMNSTHNNSSVNNNFNKMNHSISEKMGKNKNDNIFSFLKSTKNNMSFDQNGRLVNSNINYMKNKNLLLCKEEQEKHTSFQSLNCNRTKNNSIQERVVYGKEINNNHNLKDINVFKYKKHEHKHGEFFNLNNMK

HL2211 GVSNQNMNERTYAENLHNMNNIHNNKFCPSSYRHTQNILNMNSTHNNSSVNNNFNKMNHSISEKMGKNKNDNIFSFL**N**STKNNMSFDQNGRLVNSNINYMKNKNLLLCKEEQEKHTSFQSLNCNRTKNNSIQERVVYGKEINNNHNLKDINVFKYKKHEHKHGEFFNLNNMK

HL2212 GVSNQNMNERTYAENLHNMNNIHNNKFCPSSYRHTQNILNMNSTHNNSSVNNNFNKMNHSISEKMGKNKNDNIFSFLKSTKNNMSFDQNGRLVNSNINYMKNKNLLLCKEEQEKHTSFQSLNCNRTKNNSIQERVVYGKEINNNHNLKDINVFKYKKHEHKHGEFFNLNNMK

HL2213 GVSNQNMNERTYAENLHNMNNIHNNKFCPSSYRHTQNILNMNSTHNNSSVNNNFNKMNHSISEKMGKNKNDNIFSFLKSTKNNMSFDQNGRLVNSNINYMKNKNLLLCKEEQEKHTSFQSLNCNRTKNNSIQERVVYGKEINNNHNLKDINVFKYKKHEHKHGEFFNLNNMK

HL2214 GVSNQNMNERTYAENLHNMNNIHNNKFCPSSYRHTQNILNMNSTHNNSSVNNNFNKMNHSISEKMGKNKNDNIFSFLKSTKNNMSFDQNGRLVNSNINYMKNKNLLLCKEEQEKHTSFQSLNCNRTKNNSIQERVVYGKEINNNHNLKDINVFKYKKHEHKHGEFFNLNNMK

HL2301 GVSNQNMNERTYAENLHNMNNIHNNKFCPSSYRHTQNILNMNSTHNNSSVNNNFNKMNHSISEKMGKNKNDNIFSFLKSTKNNMSFDQNGRLVNSNINYMKNKNLLLCKEEQEKHTSFQSLNCNRTKNNSIQERVVYGKEINNNHNLKDINVFKYKKHEHKHGEFFNLNNMK

HL2302 GVSNQNMNERTYAENLHNMNNIHNNKFCPSSYRHTQNILNMNSTHNNSSVNNNFNKMNHSISEKMGKNKNDNIFSFLKSTKNNMSFDQNGRLVNSNINYMKNKNLLLCKEEQEKHTSFQSLNCNRTKNNSIQERVVYGKEINNNHNLKDINVFKYKKHEHKHGEFFNLNNMK

HL2303 GVSNQNMNERTYAENLHNMNNIHNNKFCPSSYRHTQNILNMNSTHNNSSVNNNFNKMNHSISEKMGKNKNDNIFSFLKSTKNNMSFDQNGRLVNSNINYMKNKNLLLCKEEQEKHTSFQSLNCNRTKNNSIQERVVYGKEINNNHNLKDINVFKYKKHEHKHGEFFNLNNMK

HL2304 GVSNQNMNERTYAENLHNMNNIHNNKFCPSSYRHTQNILNMNSTHNNSSVNNNFNKMNHSISEKMGKNKNDNIFSFLKSTKNNMSFDQNGRLVNSNINYMKNKNLLLCKEEQEKHTSFQSLNCNRTKNNSIQERVVYGKEINNNHNLKDINVFKYKKHEHKHGEFFNLNNMK

HL2305 GVSNQNMNERTYAENLHNMNNIHNNKFCPSSYRHTQNILNMNSTHNNSSVNNNFNKMNHSISEKMGKNKNDNIFSFLKSTKNNMSFDQNGRLVNSNINYMKNKNLLLCKEEQEKHTSFQSLNCNRTKNNSIQERVVYGKEINNNHNLKDINVFKYKKHEHKHGEFFNLNNMK

HL2306 GVSNQNMNERTYAENLHNMNNIHNNKFCPSSYRHTQNILNMNSTHNNSSVNNNFNKMNHSISEKMGKNKNDNIFSFLKSTKNNMSFDQNGRLVNSNINYMKNKNLLLCKEEQEKHTSFQSLNCNRTKNNSIQERVVYGKEINNNHNLKDINVFKYKKHEHKHGEFFNLNNMK

HL2307 GVSNQNMNERTYAENLHNMNNIHNNKFCPSSYRHTQNILNMNSTHNNSSVNNNFNKMNHSISEKMGKNKNDNIFSFLKSTKNNMSFDQNGRLVNSNINYMKNKNLLLCKEEQEKHTSFQSLNCNRTKNNSIQERVVYGKEINNNHNLKDINVFKYKKHEHKHGEFFNLNNMK

HL2308a GVSNQNMNERTYAENLHNMNNIHNNKFCPSSYRHTQNILNMNSTHNNSSVNNNFNKMNHSISEKMGKNKNDNIFSFLKSTKNNMSFDQNGRLVNSNINYMKNKNLLLCKEEQEKHTSFQSLNCNRTKNNSIQERVVYGKEINNNHNLKDINVFKYKKHEHKHGEFFNLNNMK

HL2308b GVSNQNMNERTYAENLHNMNNIHNNKFCPSSYRHTQNILNMNSTHNNSSVNNNFNKMNHSISEKMGKNKNDNIFSFLKSTKNNMSFDQNGRLVNSNINYMKNKNLLLCKEEQEKHTSFQSLNCNRTKNNSIQERVVYGKEINNNHNLKDINVFKYKKHEHKHGEFFNLNNMK

HL2309 GVSNQNMNERTYAENLHNMNNIHNNKFCPSSYRHTQNILNMNSTHNNSSVNNNFNKMNHSISEKMGKNKNDNIFSFLKSTKNNMSFDQNGRLVNSNINYMKNKNLLLCKEEQEKHTSFQSLNCNRTKNNSIQERVVYGKEINNNHNLKDINVFKYKKHEHKHGEFFNLNNMK

*****************************************************************************:**************************************.***************************:***************************

3D7 YPLYGKNKNIMDDDNLGNNIFHPKKKNKDEFIGSFKNNSSYVINDEDDEHYISYDDMFRNYDSDDDSNISNSKNTSENFNVKDFITNLHFANLDDDNNIISKNFFSTSKKLNDQKGEQKGEQNGEQ----KCEQ------------KYEQKYEHQGSSVKIQNNKIINKMKYD

HL1211 YPLYGKNKNIMDDDNLGNNIFHPKKKNKDEFIGSFKNNSSYVINDEDDEHYISYDDMFRNYDSDDDSNISNSKNTSENFNVKDFITNLHFANLDDDNNIISKNFFSTSKKLNDQKGEQKGEQNGEQ----KCEQ--------KYEQKYEQKYEHQGSSVKIQNNKIINKMKYD

HL1601 YPLYGKNKNIMDDDNLGNNIFHPKKKNKDEFIGSFKNNSSYVINDEDDEHYISYDDMFRNYDSDDDSNISNSKNTSENFNVKDFITNLHFANLDDDNNIISKNFFSTSKKLNDQKGEQKGEQNGEQ----KCEQ--------KYEQKYEQKYEHQGSSVKIQNNKIINKMKYD

HL1901 YPLYGKNKNIMDDDNLGNNIFHPKKKNKDEFIGSFKNNSSYVINDEDDEHYISYDDMFRNYDSDDDSNISNSKNTSENFNVKDFITNLHFANLDDDNNIISKNFFSTSKKLNDQKGEQKGEQNGEQ----KCEQ--------KYEQKYEQKYEHQGSSVKIQNNKIINKMKYD

HL1902 YPLYGKNKNIMDDDNLGNNIFHPKKKNKDEFIGSFKNNSSYVINDEDDEHYISYDDMFRNYDSDDDSNISNSKNTSENFNVKDFITNLHFANLDDDNNIISKNFFSTSKKLNDQKGEQKGEQ--------KCEQKCEQ--------KYEQKYEHQGSSVKIQNNKIINKMKYD

HL1903 YPLYGKNKNIMDDDNLGNNIFHPKKKNKDEFIGSFKNNSSYVINDEDDEHYISYDDMFRNYDSDDDSNISNSKNTSENFNVKDFITNLHFANLDDDNNIISKNFFSTSKKLNDQKGEQKGEQNGEQ----KCEQ--------KYEQKYEQKYEHQGSSVKIQNNKIINKMKYD

HL1904 YPLYGKNKNIMDDDNLGNNIFHPKKKNKDEFIGSFKNNSSYVINDEDDEHYISYDDMFRNYDSDDDSNISNSKNTSENFNVKDFITNLHFANLDDDNNIISKNFFSTSKKLNDQKGEQKGEQNGEQ----KCEQ------------KYEQKYEHQGSSVKIQNNKIINKMKYD

HL2000 YPLYGKNKNIMDDDNLGNNIFHPKKKNKDEFIGSFKNNSSYVINDEDDEHYISYDDMFRNYDSDDDSNISNSKNTSKNFNVKDFITNLHFANLDDDNNIISKNFFSTSKKLNDQKGEQKGEQNGEQ----KCEQ------------KYEQKYEHQGSSVKIQNNKIINKMKYD

HL2001 YPLYGKNKNIMDDDNLGNNIFHPKKKNKDEFIGSFKNNSSYVINDEDDEHYISYDDMFRNYDSDDDSNISNSKNTSENFNVKDFITNLHFANLDDDNNIISKNFFSTSKKLNDQKGEQKGEQNGEQ----KCEQ------------KYEQKYEHQGSSVKIQNNKIINKMKYD

HL2002 YPLYGKNKNIMDDDNLGNNIFHPKKKNKDEFIGSFKNNSSYVINDEDDEHYISYDDMFRNYDSDDDSNISNSKNTSENFNVKDFITNLHFANLDDDNNIISKNFFSTSKKLNDQKGEQKGEQNGEQ----KCEQKCEQ--------KYEQKYEHQGSSVKIQNNKIINKMKYD

HL2004 YPLYGKNKNIMDDDNLGNNIFHPKKKNKDEFIGSFKNNSSYVINDEDDEHYISYDDMFRNYDSDDDSNISNSKNTSENFNVKDFITNLHFANLDDDNNIISKNFFSTSKKLNDQKGEQKGEQNGEQ----KCEQKCEQ--------KYEQKYEHQGSSVKIQNNKIINKMKYD

HL2102 YPLYGKNKNIMDDDNLGNNIFHPKKKNKDEFIGSFKNNSSYVINDEDDEHYISYDDMFRNYDSDDDSNISNSKNTSENFNVKDFITNLHFANLDDDNNIISKNFFSTSKKLNDQKGEQKGEQNGEQ----KCEQKCEQ--------KYEQKYEHQGSSVKIQNNKIINKMKYD

HL2103 YPLYGKNKNIMDDDNLGNNIFHPKKKNKDEFIGSFKNNSSYVINDEDDEHYISYDDMFRNYDSDDDSNISNSKNTSENFNVKDFITNLHFANLDDDNNIISKNFFSTSKKLNDQKGEQKGEQNGEQ----KCEQ------------KCEQKYEHQGSSVKIQNNKIINKMKYD

HL2104 YPLYGKNKNIMDDDNLGNNIFHPKKKNKDEFIGSFKNNSSYVINDEDDEHYISYDDMFRNYDSDDDSNISNSKNTSENFNVKDFITNLHFANLDDDNNIISKNFFSTSKKLNDQKGEQKGEQNGEQ----KCEQ------------KYEQKYEHQGSSVKIQNNKIINKMKYD

HL2105 YPLYGKNKNIMDDDNLGNNIFHPKKKNKDEFIGSFKNNSSYVINDEDDEHYISYDDMFRNYDSDDDSNISNSKNTSENFNVKDFITNLHFANLDDDNNIISKNFFSTSKKLNDQKGEQKGEQNGEQ----KCEQ------------KYEQKYEHQGSSVKIQNNKIINKMKYD

HL2201 YPLYGKNKNIMDDDNLGNNIFHPKKKNKDEFIGSFKNNSSYVINDEDDEHYISYDDMFRNYDSDDDSNISNSKNTSENFNVKDFITNLHFANLDDDNNIISKNFFSTSKKLNDQKGEQKGEQNGEQ----KCEQKCEQ--------KYEQKYEHQGSSVKIQNNKIINKMKYD

HL2202 YPLYGKNKNIMDDDNLGNNIFHPKKKNKDEFIGSFKNNSSYVINDEDDEHYISYDDMFRNYDSDDDSNISNSKNTSENFNVKDFITNLHFANLDDDNNIISKNFFSTSKKLNDQKGEQKGEQNGEQ----KCEQKCEQ--------KYEQKYEHQGSSVKIQNNKIINKMKYD

HL2203 YPLYGKNKNIMDDDNLGNNIFHPKKKNKDEFIGSFKNNSSYVINDEDDEHYISYDDMFRNYDSDDDSNISNSKNTSENFNVKDFITNLHFANLDDDNNIISKNFFSTSKKLNDQKGEQKGEQNGEQNGEQKCEQ--------KYEQKYEQKYEHQGSSVKIQNNKIINKMKYD

HL2204 YPLYGKNKNIMDDDNLGNNIFHPKKKNKDEFIGSFKNNSSYVINDEDDEHYISYDDMFRNYDSDDDSNISNSKNTSENFNVKDFITNLHFANLDDDNNIISKNFFSTSKKLNDQKGEQKGEQNGEQ----KCEQ------------KYEQKYEHQGSSVKIQNNKIINKMKYD

HL2205 YPLYGKNKNIMDDDNLGNNIFHPKKKNKDEFIGSFKNNSSYVINDEDDEHYISYDDMFRNYDSDDDSNISNSKNTSENFNVKDFITNLHFANLDDDNNIISKNFFSTSKKLNDQKGEQKGEQNGEQ----KCEQ--------KYEQKYEQKYEHQGSSVKIQNNKIINKMKYD

HL2206 YPLYGKNKNIMDDDNLGNNIFHPKKKNKDEFIGSFKNNSSYVINDEDDEHYISYDDMFRNYDSDDDSNISNSKNTSENFNVKDFITNLHFANLDDDNNIISKNFFSTSKKLNDQKGEQKGEQNGEQ----KCEQ--------KYEQKYEQKYEHQGSSVKIQNNKIINKMKYD

HL2208 YPLYGKNKNIMDDDNLGNNIFHPKKKNKDEFIGSFKNNSSYVINDEDDEHYISYDDMFRNYDSDDDSNISNSKNTSENFNVKDFITNLHFANLDDDNNIISKNFFSTSKKLNDQKGEQKGEQNGEQ----KCEQ------------KYEQKYEHQGSSVKIQNNKIINKMKYD

HL2209 YPLYGKNKNIMDDDNLGNNIFHPKKKNKDEFIGSFKNNSSYVINDEDDEHYISYDDMFRNYDSDDDSNISNSKNTSENFNVKDFITNLHFANLDDDNNIISKNFFSTSKKLNDQKGEQKGEQNGEQNGEQKCEQ--------KYEQKYEQKYEHQGSSVKIQNNKIINKMKYD

HL2210 YPLYGKNKNIMDDDNLGNNIFHPKKKNKDEFIGSFKNNSSYVINDEDDEHYISYDDMFRNYDSDDDSNISNSKNTSENFNVKDFITNLHFANLDDDNNIISKNFFSTSKKLNDQKGEQKGEQNGEQ----KCEQ------------KYEQKYEHQGSSVKIQNNKIINKMKYD

HL2211 YPLYGKNKNIMDDDNLGNNIFHPKKKNKDKFIGSFKNNSSYVINDEDDEHYISYDDMFRNYDSDDDSNISNSKNTSENFNVKDFITNLHFANLDDDNNIISKNFFSTSKKLNDQKGEQKGEQNGEQ----KCEQKCEQKCEQ----KYEQKYEHQGSSVKIQNNKIINKMKYD

HL2212 YPLYGKNKNIMDDDNLGNNIFHPKKKNKDEFIGSFKNNSSYVINDEDDEHYISYDDMFRNYDSDDDSNISNSKNTSENFNVKDFITNLHFANLDDDNNIISKNFFSTSKKLNDQKGEQKGEQNGEQ----KCEQ------------KYEQKYEHQGSSVKIQNNKIINKMKYD

HL2213 YPLYGKNKNIMDDDNLGNNIFHPKKKNKDEFIGSFKNNSSYVINDEDDEHYISYDDMFRNYDSDDDSNISNSKNTSENFNVKDFITNLHFANLDDDNNIISKNFFSTSKKLNDQKGEQKGEQNGEQ----KCEQ------------KYEQKYEHQGSSVKIQNNKIINKMKYD

HL2214 YPLYGKNKNIMDDDNLGNNIFHPKKKNKDEFIGSFKNNSSYVINDEDDEHYISYDDMFRNYDSDDDSNISNSKNTSENFNVKDFITNLHFANLDDDNNIISKNFFSTSKKLNDQKGEQKGEQNGEQ----KCEQ------------KYEQKYEHQGSSVKIQNNKIINKMKYD

HL2301 YPLYGKNKNIMDDDNLGNNIFHPKKKNKDEFIGSFKNNSSYVINDEDDEHYISYDDMFRNYDSDDDSNISNSKNTSENFNVKDFITNLHFANLDDDNNIISKNFFSTSKKLNDQKGEQKGEQNGEQ----KCEQKCEQ--------KYEQKYEHQGSSVKIQNNKIINKMKYD

HL2302 YPLYGKNKNIMDDDNLGNNIFHPKKKNKDEFIGSFKNNSSYVINDEDDEHYISYDDMFRNYDSDDDSNISNSKNTSENFNVKDFITNLHFANLDDDNNIISKNFFSTSKKLNDQKGEQ----NGEQ----KCEQ--------KYEQKYEQKYEHQGSSVKIQNNKIINKMKYD

HL2303 YPLYGKNKNIMDDDNLGNNIFHPKKKNKDEFIGSFKNNSSYVINDEDDEHYISYDDMFRNYDSDDDSNISNSKNTSENFNVKDFITNLHFANLDDDNNIISKNFFSTSKKLNDQKGEQKGEQNGEQNGEQKCEQ--------KYEQKYEQKYEHQGSSVKIQNNKIINKMKYD

HL2304 YPLYGKNKNIMDDDNLGNNIFHPKKKNKDEFIGSFKNNSSYVINDEDDEHYISYDDMFRNYDSDDDSNISNSKNTSENFNVKDFITNLHFANLDDDNNIISKNFFSTSKKLNDQKGEQKGEQNGEQ----KCEQ--------KYEQKYEQKYEHQGSSVKIQNNKIINKMKYD

HL2305 YPLYGKNKNIMDDDNLGNNIFHPKKKNKDEFIGSFKNNSSYVINDEDDEHYISYDDMFRNYDSDDDSNISNSKNTSENFNVKDFITNLHFANLDDDNNIISKNFFSTSKKLNDQKGEQKGEQNGEQ----KCEQ--------KYEQKYEQKYEHQGSSVKIQNNKIINKMKYD

HL2306 YPLYGKNKNIMDDDNLGNNIFHPKKKNKDEFIGSFKNNSSYVINDEDDEHYISYDDMFRNYDSDDDSNISNSKNTSENFNVKDFITNLHFANLDDDNNIISKNFFSTSKKLNDQKGEQKGEQKGEQNGEQKCEQ------------KYEQKYEHQGSSVKIQNNKIINKMKYD

HL2307 YPLYGKNKNIMDDDNLGNNIFHPKKKNKDEFIGSFKNNSSYVINDEDDEHYISYDDMFRNYDSDDDSNISNSKNTSENFNVKDFITNLHFANLDDDNNIISKNFFSTSKKLNDQKGEQKGEQNGEQNGEQKCEQ--------KYEQKYEQKYEHQGSSVKIQNNKIINKMKYD

HL2308 YPLYGKNKNIMDDDNLGNNIFHPKKKNKDEFIGSFKNNSSYVINDEDDEHYISYDDMFRNYDSDDDSNISNSKNTSENFNVKDFITNLHFANLDDDNNIISKNFFSTSKKLNDQKGEQKGEQNGEQNGEQKCEQ--------KYEQKYEQKYEHQGSSVKIQNNKIINKMKYD

HL2309 YPLYGKNKNIMDDDNLGNNIFHPKKKNKDEFIGSFKNNSSYVINDEDDEHYISYDDMFRNYDSDDDSNISNSKNTSENFNVKDFITNLHFANLDDDNNIISKNFFSTSKKLNDQKGEQKGEQNGEQ----KCEQ--------KYEQKYEQKYEHQGSSVKIQNNKIINKMKYD

********************************************************************************************************************** **** **** ***************************

**Suppl. Fig. 3. Clustal omega alignment of UBP-1 fragment III amino acids encoded by codons 2519 – 2847 of the *pfubp1* gene from 37 parasite lines evaluated in this study.**

3D7_ref NHVNRMDGVNHVNRMDGVNHVNRMDGVNRVN------------------RMNHANRVSRMNHANRVSRMNHANRVSRMNHANRVSPNNIEDIRMGGVKIKKYLMLPINKFTFENM

HL1211 NHVNRMDGVNHVNRMDGVNHVNRMDGVNRVN---------------------------RMNHANRVSRMNHANRV**N**RMNHANRVSPNNIEDIRMGGVKIKKYLMLPINKFTFENM

HL1402 NHVNRMDGVNHVNRMDGVNHVNRMDGVNRVN------------------RMNHANRVSRMNHANRVSRMNHANRVSRMNHANRVSPNNIEDIRMGGVKIKKYLMLPINKFTFENM

HL1601 NHVNRMDGVNHVNRMDGVNHVNRMDGVNRVN---------------------------RMNHANRVSRMNHANRVSRMNHANRVSPNNIEDIRMGGVKIKKYLMLPINKFTFENM

HL1601 NHVNRMDGVNHVN---------RMDGVNRVN---------------------------RMNHANRVSRMNHANRVSRMNHANRVSPNNIEDIRMGGVKIKKYLMLPINKFTFENM

HL2102 NHVNRMDGVNHVNRMDGVNHVNRMDGVNRVN------------------------------------RMNHANRVSRMNHANRVSPNNIEDIRMGGVKIKKYLMLPINKFTFENM

HL2104 NHVNRMDGVNHVNRMDGVNHVNRMDGVNRVN---------------------------RMNHANRVSRMNHANRVSRMNHANRVSPNNIEDIRMGGVKIKKYLMLPINKFTFENM

HL2201 NHVNRMDGVNHVNRMDGVNHVNRMDGVNRVN------------------RMNHANRVSRMNHANRVSRMNHANRVSRMNHANRVSPNNIEDIRMGGVKIKKYLMLPINKFTFENM

HL2202 NHVNRMDGVNHVNRMDGVNHVNRMDGVNRVN---------RMNHANRVN---------RMNHANRVSRMNHANRVSRMNHANRVSPNNIEDIRMGGVKIKKYLMLPINKFTFENM

HL2203 NHVNRMDGVNHVNRMDGVNHVNRMDGVNRVN---------------------------RMNHANRVSRMNHANRVSRMNHANRVSPNNIEDIRMGGVKIKKYLMLPINKFTFENM

HL2204 NHVNRMDGVNHVNRMDGVNHVNRMDGVNRVN---------------------------RMNHANRVSRMNHANRVSRMNHANRVSPNNIEDIRMGGVKIKKYLMLPINKFTFENM

HL2205 NHVNRMDGVNHVNRMDGVNHVNRMDGVNRVN------------------RMNHANRVSRMNHANRVSRMNHANRVSRMNHANRVSPNNIEDIRMGGVKIKKYLMLPINKFTFENM

HL2206 NHVNRMDGVNHVNRMDGVNHVNRMDGVNRVN---------------------------RMNHANRVSRMNHANRVSRMNHANRVSPNNIEDIRMGGVKIKKYLMLPINKFTFENM

HL2208 NHVNRMDGVNHVNRMDGVNHVNRMDGVNRVN---------------------------RMNHANRVS**L**MNHANRVSRMNHANRVSPNNIEDIRMGGVKIKKYLMLPINKFTFENM

HL2209 NHVNRMDGVNHVNRMDGVNHVNRMDGVNRVN---------RMNHANRVN------------------RMNHANRVSRMNHANRVSPNNIEDIRMGGVKIKKYLMLPINKFTFENM

HL2210 NHVNRMDGVNHVNRMDGVNHVNRMDGVNRVN------------------RMNHANRVSRMNHANRVSRMNHANRVSRMNHANRVSPNNIEDIRMGGVKIKKYLMLPINKFTFENM

HL2212 NHVNRMDGVNHVN------------------RMDGVNRVS---------------------------RMNHANRVSRMNHANRVSPNNIEDIRMGGVKIKKYLMLPINKFTFENM

HL2213 NHVNRMDGVNHVNRMDGVNHVNRMDGVNRVN---------------------------RMNHANRVSRMNHANRVSRMNHANRVSPNNIEDIRMGGVKIKKYLMLPINKFTFENM

**Suppl. Fig. 4. Clustal omega alignment of UBP-1 fragment IV amino acids encoded by codons 3064-3160 of the *pfubp1* gene from 17 parasite lines evaluated in this study.**
